# Validation of the Computerized Pediatric Triage Tool, *pediaTRI*, in the Pediatric Emergency Department of Lenval Children's Hospital in Nice: A Cross-Sectional Observational Study

**DOI:** 10.3389/fped.2022.840181

**Published:** 2022-04-26

**Authors:** Antoine Tran, Petri Valo, Camille Rouvier, Emmanuel Dos Ramos, Emma Freyssinet, Emma Baranton, Olivier Haas, Hervé Haas, Christian Pradier, Stéphanie Gentile

**Affiliations:** ^1^Pediatric Emergency Department, Lenval University Children's Hospital, Nice, France; ^2^School of Medicine, Université Côte d'Azur, Nice, France; ^3^Research Team EA 3279 “Santé Publique, Maladies Chroniques et Qualité de Vie”, School of Medicine, Aix-Marseille Université, Marseille, France; ^4^School of Computing, University of Eastern Finland, Joensuu, Finland; ^5^Department of Medical Computing, General Hospital “les Palmiers”, Hyères, France; ^6^Innovation e-Santé Sud, Groupement d'Intérêt Public, Hyères, France; ^7^Department of Pediatrics, Centre Hospitalier Princesse-Grace, Monaco, Monaco; ^8^Department of Public Health, Archet University Hospital, Nice, France

**Keywords:** pediatric triage tool, validation, performance, reference standard, pediatric early warning score, pediatric emergency department

## Abstract

**Introduction:**

A reliable pediatric triage tool is essential for nurses working in pediatric emergency departments to quickly identify children requiring priority care (high-level emergencies) and those who can wait (low-level emergencies). In the absence of a gold standard in France, the objective of our study was to validate our 5-level pediatric triage tool –*pediaTRI*– against the reference tool: the Pediatric Early Warning Score (PEWS) System.

**Materials and Methods:**

We prospectively included 100,506 children who visited the Pediatric Emergency Department at Lenval Children's Hospital (Nice, France) in 2016 and 2017. The performance of *pediaTRI* to identify high-level emergencies (severity levels 1 and 2) was evaluated in comparison with a PEWS ≥ 4/9. Data from 2018–19 was used as an independent validation cohort.

**Results:**

*pediaTRI* agreed with the PEWS score for 84,896 of the patients (84.5%): 15.0% (14.8–15.2) of the patients were over-triaged and 0.5% (0.5–0.6) under-triaged compared with the PEWS score. *pediaTRI* had a sensitivity of 76.4% (74.6–78.2), a specificity of 84.7% (84.4–84.9), and positive and negative likelihood ratios of 5.0 (4.8–5.1) and 0.3 (0.3–0.3), respectively, for the identification of high-level emergencies. However, the positive likelihood ratios were lower for patients presenting with a medical complaint [4.1 (4.0–4.2) v 10.4 (7.9–13.7 for trauma), and for younger children [1.2 (1.1–1.2) from 0 to 28 days, and 1.9 (1.8–2.0) from 28 days to 3 months].

**Conclusion:**

*pediaTRI* has a moderate to good validity to triage children in a Pediatric Emergency Department with a tendency to over-triage compared with the PEWS system. Its validity is lower for younger children and for children consulting for a medical complaint.

## Introduction

The number of visits to emergency departments (ED) has been rising steadily for both adult and pediatric patients over the past decades (1) resulting in an increase in waiting and care times. Each ED manages a wide variety of pathologies ranging from a simple general consultation to a life-threatening emergency. However, overcrowding in the ED as well as difficulties in monitoring patients waiting for clinical examination, can endanger patient safety (2). Patients require prioritization and triaging as soon as they reach the ED and cannot be seen purely in the order of arrival. An ideal triage system should be able to identify those who require immediate care (high-level emergency) from those who can wait or those who will not require emergency care (intermediate- to low-level emergency) (3). This triage is mostly carried out by a nurse at the triage zone (4) who must quickly identify high-emergency patients requiring immediate care and organize their care pathway. The triage nurse uses a decision support tool known as a triage tool.

There are many triage tools around the world, all of which have five levels of triage ranging from a non-urgent consultation (Level 5) to a life-threatening emergency (Level 1) (5–9). However, these tools are not suitable in all EDs because they rely on the diagnostic skills of a “clinical” nurse, a qualification that does not exist in all countries, including France. In addition, these tools were initially designed for adult patients, and then adapted to the pediatric patient, while a child cannot be considered as “a miniature adult.”

Regardless of the triage algorithm, the identification of life-threatening emergencies is a common and implicit denominator. In a pediatric ED (PED) setting, a high-level emergency corresponds to a child presenting an immediate life-threatening risk that could lead to cardio-respiratory arrest or a related emergency, and thus requires rapid intervention. These patients, for whom a Level 1 or 2 is usually assigned by commonly used pediatric triage tools (10–13), can also be screened using warning scores (14–16) that are predictive of clinical deterioration within 24 hours after visiting the PED. Among them, the Pediatric Early Warning System (PEWS) system, created in 2001, is considered to be efficient, easy to use, and reliable (17–21).

In France, there is no gold standard in pediatric triage and each hospital uses their own “home-made” triage system. Portas et al. (22) aimed to validate their pediatric triage tool correlating treatment times with severity and the hospitalization prediction rate. However, this was a 3-level scale that excluded newborns and trauma complaints, and the validity study was based on the correlation of resource utilization rather than comparing the triage level against a reference standard.

In 2000, the PED of the University Hospital of Nice (France) created a 5-level pediatric triage tool – the *pediaTRI –* based on clinical items of inspection, interview, and analysis of vital signs (Appendix 1). The items were initially selected by a panel of local experts based on the most life-threatening conditions (i.e., purpura fulminans, meningitis, severe pediatric trauma) and the most frequent diseases (i.e., bronchiolitis, gastroenteritis, traumatic brain injury). In 2011, the tool was integrated into the pediatric triage module of the Terminal Urgences® (TU®) (23), a software developed by the Regional Emergency Observatory of the South East of France (ORU Provence Alpes Côte d'Azur, France) under the supervision of the Regional Health Agency (ARS). However, this computerized version of *pedia*TRI has never been evaluated.

Our objective was to validate *pediaTRI* integrated into the TU® software against a reference standard, the PEWS system, in screening high-level emergencies.

## Methodology

### Design

We conducted a cross-sectional observational study in the PED of the Lenval Children's Hospital in Nice, a tertiary level, University Teaching Children's Hospital. This PED is the only one in the southeast of France (1.140.000 inhabitants aged 0 to 19 in 2017) and is the 4th biggest PED in France with an average number of 60.000 visits per year (before the Covid 19 Pandemic) of patients from zero to 17 years of age. The hospital is a reference center for specific diseases including cystic fibrosis, neuromuscular diseases, sudden infant death syndrome, and psycho-trauma, and provides a complete medico-surgical and technical platform delivering 24/7 care for any emergency (medical, surgical, psychiatric) with two operating rooms managed by pediatric anesthesiologists and surgeons. It has 180 beds including 10 belonging to the Intensive Care Unit (ICU) and 60 for outpatient care. The patient recruitment pool covers a radius of 90 miles from Nice. Although our PED is referenced as one of the two Level 1 pediatric trauma centers in the area, potentially life- threatening conditions remain rare and represent about 1% of the visits per year.

### Participants

All patients under the age of 18 who visited the PED of Lenval Children's Hospital between January 1, 2016 and December 31, 2017 (open 24/7) were included. Patients who were called back, patients treated in prehospital care, and patients who left without an assigned triage level were not included. Patients visiting the PED for psychological or psychiatric conditions, patients who left without being seen by a physician, whose recorded vital signs were considered as uninterpretable (not reliable or error measures), or for whom there was no information about outcome, were excluded. The study was approved by the French Data Protection Authority (CNIL) (registration number: 2157640v0) in accordance with the laws which govern “non-interventional clinical research” in France (namely articles L.1121-1 and R.1121-2 of the public health code). Patient data were anonymized using a specific patient numbering procedure for the study. Informed written consent or ethics committee authorization was not necessary for this no-effect observational study collecting anonymized data on patient management.

### The Pediatric Triage Tool Integrated Into the Terminal Urgences® Software

The Terminal Urgences® software was created in 2002 under the supervision of the Regional Health Agency, and is now used in more than 65 EDs (for both adult and pediatric patients) in the southeast of France (24). This software contains many modules – a triage module, medical file module (medical and paramedical), nursing care prescription module (procedures and care), and a therapeutic prescription module – which are accessed and completed in real time by various health professionals.

In pediatric triage, the nurse follows a pre-established evaluation algorithm for triaging patients. Firstly, the nurse must identify whether the patient is in a critical condition (e.g., in respiratory or cardiac failure) or judged to be at high risk (e.g., newborns, immunosuppressed). In those cases, the triage process is stopped at this point so that lifesaving care can be initiated. For the other children, the nurse collects details about the chief complaint covering all the triage items, and selects all the complaints that the child presents or that are reported by the parent(s). Each item is assigned to a triage level, although the triage can be moderated by risky situations/conditions and out-of-range values for vital signs. If several items are selected, the one with the highest level of severity determines the final triage level. If the nurse disagrees with the level assigned by the tool, they can change the triage level after consulting with the attending PED physician. The triage module is a user-friendly interface allowing the nurse to perform a quick triage within an optimal duration of 5 min.

The pediatric triage tool, *pediaTRI*, is structured into five levels ranging from the most severe (Level 1) to the least severe (Level 5). Levels 1 (life-threatening emergency) and 2 (very urgent) are mainly assigned to patients for whom the vital and / or functional prognosis has already been engaged or will probably be engaged very quickly if no care is administered. These patients are defined as high-level emergencies. Low-level emergencies correspond to Levels 3 (urgent), 4 (consultation), and 5 (non-urgent) for which a vital or functional prognosis may not be engaged either in the short or medium term. However, according to resource utilization and to the architecture of the PED at Lenval Children's Hospital, some risky situations (i.e., newborns) or risky profiles (i.e., immunosuppressed patients) may be systematically attributed Level 2. Each triage level determines the optimal time (in min) for the patient to see a physician (fractile response) (25) according to the organization of the PED: 0, 15, 60, 120, or 240 min, respectively, for triage levels 1 to 5.

All the nurses must have worked in the PED for at least 6 months and then undergone a 3-month supervision period by an experienced nurse before performing a triage alone.

### Justification of the Reference Standard

We could not validate *pediaTRI* against other triage tools developed in this setting as the quality of evidence (robust validation) to support their use is poor.

Among the existing scores for predicting mortality and morbidity in pediatrics, we opted for the PEWS system described by Akre et al. (26) as our reference standard due to its simplicity of use based on clinical signs for triage. PEWS was created in the UK in 2001 specifically for children (rather than an adult tool adapted to pediatrics) by a panel of experts to rapidly detect clinical deterioration including cardiopulmonary arrest (CPA) by the use of a simple and objective clinical tool. Studies have shown that PEWS identifies pediatric patients who require intensive care (16, 27). The PEWS system is based on three main components each given a 3-point rating as follows: (a) behavior and early signs of shock, recognizable and assessable by the parents; (b) skin tone and capillary refill time to assess the cardiovascular system; (c) and respiratory rate and oxygen dependence to assess the respiratory system. According to the literature (26–28), the optimal cutoff level to calculate the sensitivity and specificity for admission to an ICU, defined as a high-level emergency, is ≥ 4/9 with a sensitivity ranging from 61.3 to 94.4% and specificity from 25.2 to 86.7% (16).

### Sample Size

Based on data from previous years, approximately 200 patients classified as Level 1 (life-threatening emergency) visit our PED each year. Thus, we conducted a large cross-sectional study to allow for sufficient statistical power and detailed evaluation of specific categories of patients by analyzing patients visiting our PED over 2 successive years (2016–17).

### Endpoints

The primary endpoint was the evaluation of the performance of *pediaTRI* in classifying patients as a high-level emergency (*pediaTRI* Levels 1 or 2) or low-level emergency (Levels 3, 4, and 5). In the same way, a PEWS score ≥ 4/9 defined a high-level emergency vs. a PEWS score ≤ 3/9 as a low-level emergency. Postoperative vomiting and aerosol administration were not assessed. The secondary endpoint was the performance of *pediaTRI* according to gender, age (divided into subgroups), type of complaint (medical or surgical), and the main diagnosis. The final endpoint was the evaluation of the markers of severity such as length of stay (LOS) and hospitalization rates.

### Data

Data from 2016–17 constituted the main dataset, and data from 2018–19 was used as an independent validation cohort. Patient data were collected prospectively in the TU® software: gender (male/female), age (recorded as age groups: 0 – 27 days, 28 days – 3 months, 3 months – 1 year, 1 – 3 years, 3 – 7 years, 7 – 12 years, and 12 – 18 years), data from *pediaTRI* with the level of triage (from 1 to 5), the PEWS score calculation, medical complaints (categorized as: ENT, pulmonary, cardiovascular, neurology, digestive, urology-nephrology, gynecology, dermatology, endocrinology-metabolism, infectious diseases, rheumatology and pain, hematology, poisoning, and others), surgical complaints (categorized as head and neck trauma, upper and lower limb trauma, trauma of the trunk-pelvis-urogenital apparatus, burns, and others), final diagnosis and LOS in mins.

The vital signs such as the pediatric Glasgow score (/15), heart rate (in number of beats per minute), respiratory rate (in number of breaths per minute), pulsed oxygen saturation (in %) with details of oxygen input or not (in ambient air or in L/min depending on the case) were collected into the triage module. The patient's outcome (hospitalization in ICU or another ward, transfer to another establishment, discharged home) was collected in the medical file module. The diagnoses were entered in the diagnosis and procedures rating module. We considered the vital signs to be normal if they were not entered by the triage nurse.

### Statistical Analysis

The data, compiled exclusively as categorical comparisons, were evaluated using the chi-squared test. Over-triage was defined as a higher *pediaTRI* triage level compared with PEWS. Conversely, under-triage corresponded to a lower triage level.

The validity of *pediaTRI* was evaluated vs. PEWS using sensitivity, specificity, positive and negative predictive values, and positive and negative likelihood ratios. The results are expressed in numbers and percentages with 95% confidence intervals. Taking into account our sample size, the significance level “*p*” was set at 0.001 to avoid random statistical significance (29) with readjustment during multiple comparisons according to the Bonferroni method. One-way analysis of variance (ANOVA) was used to determine mean differences among patients for LOS in the PED, and post hoc comparisons were conducted using Scheffe's post hoc tests. Missing data were not analyzed as they represented 6% of the study sample only with negligible impact on our final outcomes. Statistical analyses were performed using R Studio version 4.0.2 for Macintosh® software from the Lenval Children's Hospital.

### Validation Cohort

We decided to conduct a single-center study due to a lack of training in pediatric triage and the use of *pediaTRI* in other centers. In 2015, the steering committee of the ORUPACA recommended evaluating the performance of *pediaTRI* at the Lenval Children's Hospital before introducing it in other PEDs of the region subject to prior training in pediatric triage. As external validation was not possible, the 2018–2019 dataset was used as an independent validation cohort (the statistical analyses for the two periods were identical and are provided in Appendix 2).

## Results

**Figure 1:**
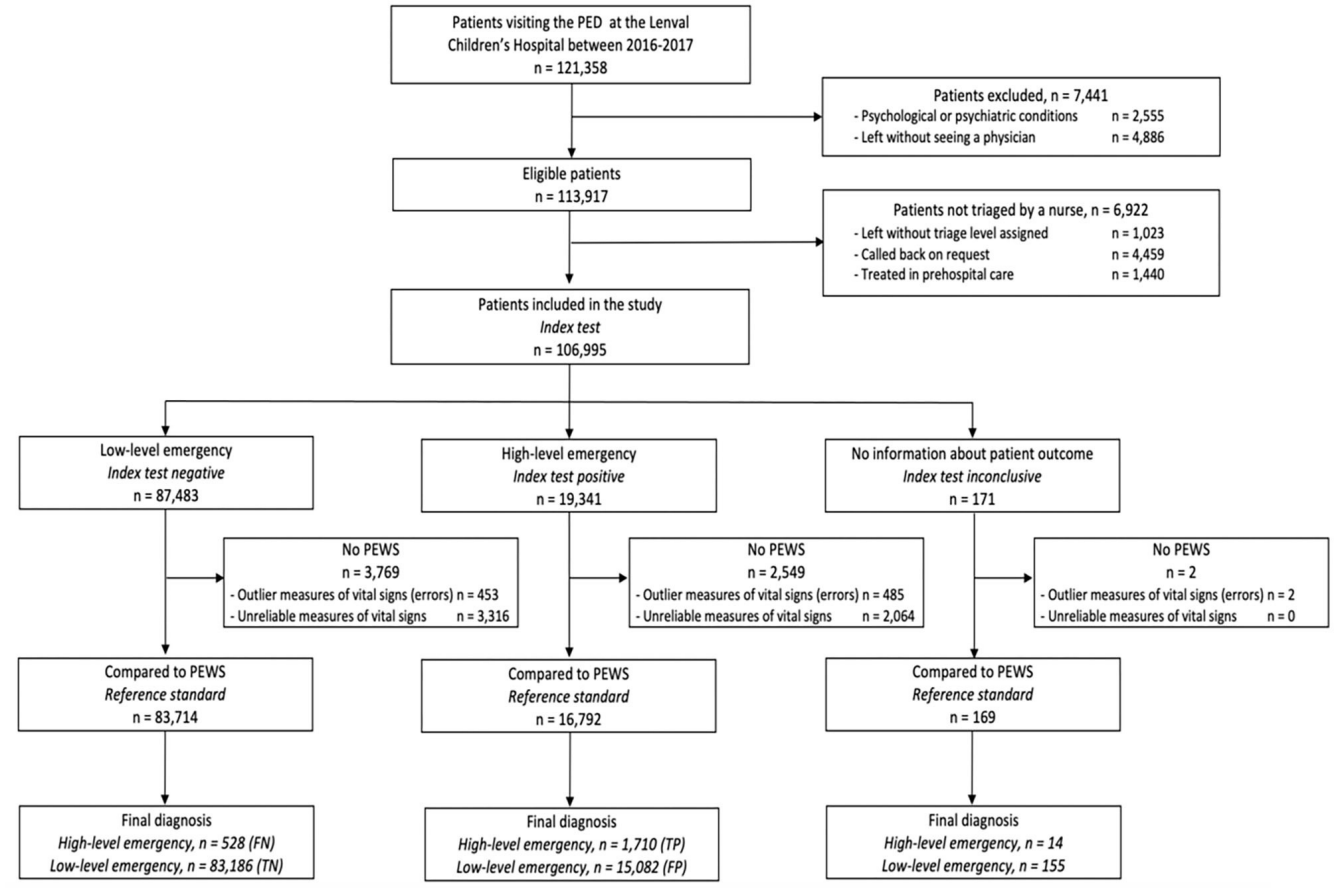
STARD flow diagram presenting patients triaged by both *pediaTRI* (index test) and PEWS (reference standard). TP, true positive; TN, true negative; FP, false positive; FN, false negative.

Among the 121,358 patients who visited the PED -between January 1, 2016 and December 31, 2017, 100,506 children were included in the analyses (82.8%), 70,330 of whom [70.0% (95% CI 69.7–70.3)] presented medical complaints (*p* < 0.001) (Figure 1).

Patients triaged as Level 4 (simple consultation) and Level 3 (urgent) were the most prevalent (Table 1): 41.1% (40.8–41.4) and 34.7% (34.4–35.0), respectively (*p* < 0.001) (Table 1). Boys were rated significantly more severe than girls: 59.9% (53.1–66.5), 59.4% (58.6–60.1) and 55.1 (54.6–55.7), respectively, for Levels 1, 2, and 3 (*p* < 0.001). Patients evaluated at Level 1 were more likely to be from 1 to 3 years old (29.0% (23.1–35.6) while those evaluated at Levels 2, 3, 4, or 5 were more likely to be 3 to 7 years old [22.2% (21.5–22.8), 29.7% (29.2–30.2), 27.2% (26.8–27.7) and 26.9% (25.9–27.9), respectively] (*p* < 0.001).

**Table 1:** Characteristics of the sample by level of triage.

		***n***	**Level 1 (***n*** =217)**	**Level 2 (***n*** =16575)**	**Level 3 (***n*** =34,832)**	**Level 4 (***n*** =41,302)**	**Level 5 (***n*** =7580)**
Total^†^		100,506	0.2 (0.2–0.2)	16.5 (16.3–16.7)	34.7 (34.4–35.0)	41.1 (40.8–41.4)	7.5 (7.4–7.7)
Sex^†^	Male	55,166	59.9 (53.1–66.5)	59.4 (58.6–60.1)	55.1 (54.6–55.7)	52.9 (52.4–53.4)	54.7 (53.5–55.8)
	Female	45,333	40.1 (33.5–46.9)	40.6 (39.9–41.4)	44.9 (44.3–45.4)	47.1 (46.6–47.6)	45.3 (44.2–46.5)
Age^†^	[0–28 d]	1,262	1.8 (0.5–4.7)	6.5 (6.1–6.9)	0.2 (0.2–0.3)	0.2 (0.2–0.2)	0.4 (0.2–0.5)
	[28 d−3 m]	2,462	2.8 (1.0–5.9)	7.9 (7.5–8.3)	1.6 (1.4–1.7)	1.2 (1.1–1.3)	1.6 (1.4–1.9)
	[3 m−1 y]	9,991	14.3 (9.9–19.7)	12.9 (12.4–13.4)	11.5 (11.1–11.8)	7.1 (6.8–7.3)	11.8 (11.1–12.5)
	[1 y−3 y]	22,622	29.0 (23.1–35.6)	21.4 (20.8–22.1)	25.9 (25.4–26.3)	20.1 (19.7–20.5)	22.6 (21.7–23.6)
	[3 y−7 y]	27,372	24.9 (19.3–31.2)	22.2 (21.5–22.8)	29.7 (29.2–30.2)	27.2 (26.8–27.7)	26.9 (25.9–27.9)
	[7 y−12 y]	21,405	14.3 (9.9–19.7)	15.6 (15.0–16.1)	18.3 (17.9–18.7)	26.1 (25.7–26.5)	21.6 (20.7–22.6)
	[12 y−18 y]	15,392	12.9 (8.7–18.1)	13.6 (13.0–14.1)	12.9 (12.5–13.2)	18.1 (17.8–18.5)	15.1 (14.3–15.9)
Outcome^†^	Intensive care unit	137	13.8 (9.5–19.1)^‡^	0.5 (0.4–0.6)^‡^	0.0 (0.0–0.1)	0.0 (0.0–0.0)	0.0 (0.0–0.1)
	Hospitalization^a^	5,838	53.5 (46.6–60.2)^‡^	21.8 (21.2–22.4)^‡^	4.8 (4.6–5.0)^‡^	1.0 (0.9–1.1)^‡^	0.6 (0.4–0.7)^‡^
	Transfer^b^	87	0.0 (0.0–1.7)	0.3 (0.3–0.4)^‡^	0.1 (0.0–0.1)	0.0 (0.0–0.1)	0.0 (0.0–0.0)
	Discharged	94,444	32.7 (26.5–39.4)^‡^	77.4 (76.7–78.0)^‡^	95.1 (94.9–95.4)^‡^	99 (98.9–99.1)^‡^	99.4 (99.2–99.6)^†^
Diagnosis	ENT infectious diseases^M†^	15,978	0.5 (0.0–2.5)	6.8 (6.4–7.2)	18.4 (18.0–18.8)	17.2 (16.8–17.5)	17.6 (16.8–18.5)
	Acute gastroenteritis^M†^	9,002	3.7 (1.6–7.1)	12.0 (11.5–12.5)	14.5 (14.1–14.9)	4.5 (4.3–4.7)	1.6 (1.3–1.9)
	Asthma^M†^	2,856	22.1 (16.8–28.2)	10.0 (9.6–10.5)	3.1 (3.0–3.3)	0.1 (0.1–0.1)	0.1 (0–0.2)
	Bronchiolitis^M†^	1,654	8.3 (5.0–12.8)	6.6 (6.2–6.9)	1.4 (1.3–1.5)	0.1 (0.1–0.2)	0.2 (0.1–0.3)
	Flu^M†^	1,669	0.5 (0.0–2.5)	1.6 (1.4–1.8)	2.5 (2.3–2.6)	1.2 (1.1–1.3)	0.8 (0.7–1.1)
	Fever^Mc†^	4,605	1.4 (0.3–4.0)	4.5 (4.2–4.8)	5.8 (5.5–6.0)	3.7 (3.5–3.9)	4.1 (3.7–4.6)
	Mild trauma brain injury^S†^	5,400	6.5 (3.6–10.6)	3.4 (3.1–3.7)	6.5 (6.3–6.8)	3.3 (3.1–3.5)	15.6 (14.8–16.4)
	Upper limb trauma^S†^	10,062	0.9 (0.1–3.3)	4.9 (4.5–5.2)	6.6 (6.3–6.9)	14.7 (14.4–15.1)	11.4 (10.7–12.1)
	Lower limb trauma^S†^	8,402	0.9 (0.1–3.3)	1.5 (1.4–1.7)	2.5 (2.3–2.7)	15.6 (15.3–16.0)	11.0 (10.3–11.7)
	Burn^S†^	461	4.6 (2.2–8.3)	0.7 (0.6–0.8)	0.6 (0.5–0.7)	0.3 (0.2–0.3)	0.2 (0.1–0.4)
	Visceral pathologies^Sd†^	1,142	0.0 (0.0–1.7)	2.8 (2.6–3.1)	1.4 (1.3–1.6)	0.4 (0.4–0.5)	0.0 (0.0–0.1)
PED LOS (min)^e†^		94,273	176 ± 124.4^§^	136.9 ± 88.9^§^	125 ± 82.6^§^	107 ± 69.7^§^	96.5 ± 63.1^§^

*Values are presented as percentages with their 95% CI and the length of stay (PED LOS) is presented as means with their standard deviation*.

† *p < 0.001 by level of triage*;

‡ *p < 0.001 according to “outcome”, post hoc comparisons by level of triage*;

§ *p < 0.001 according to “PED LOS”, post hoc comparisons by level of triage*.

*M, Top 6 of the medical diagnosis; S, Top 5 of the surgical diagnosis*.

a *Medical and surgical units*.

b *87 children were transferred to 2 pediatric units (hematology, neonatology) that are located at the Archet university hospital of Nice (2miles away from the Lenval Children's hospital)*.

c *Fever of unknown origin*.

d *Appendicitis, occlusive pathologies, hernial pathologies, ovarian pathologies*.

e *Length of stay defined as the duration between the time of the admission and the time of the exit of the PED (in minutes)*.

The most common presentations for a medical complaint (Table 2) were:

Level 1: Pulmonary [42.9% (36.2–49.7)] and neurological [18.0% (13.1–23.7)] conditions
Level 2: Pulmonary [31.0 (30.3–31.7)] and digestive [21.2% (20.6–21.8)] conditions
Level 3: Digestive (23.8% (23.3–24.2) and pulmonary conditions [18.1% (17.7–18.5)], and infectious diseases [15.4 (15.0–15.8)]
Levels 4 and 5: Dermatological [12.3% (11.9–12.6) and 20.6% (19.7–21.6)], ENT conditions [14.8% (14.4–15.1) and 14.6% (13.8–15.4)], and infectious diseases [12.2% (11.9–12.6)] and 15.3% (14.5–16.1)] for Levels 4 and 5, respectively (*p* < 0.001).

**Table 2:** Characteristics of complaints by level of triage.

		***n***	**Level 1 (***n*** =217)**	**Level 2 (***n*** =16,575)**	**Level 3 (***n*** =34,832)**	**Level 4 (***n*** =41,302)**	**Level 5 (***n*** =7,580)**
Type of complaint^†^	Medical	70,330	82.5 (76.8–87.3)	86.4 (85.9–87.0)	75.8 (75.4–76.3)	61.1 (60.6–61.6)	55.2 (54.1–56.3)
	Surgical	30,176	17.5 (12.7–23.2)	13.6 (13.0–14.1)	24.2 (23.7–24.6)	38.9 (38.4–39.4)	44.8 (43.7–45.9)
Medical complaint	ENT diseases^†^	9,200	1.8 (0.5–4.7)	3.0 (2.7–3.3)	4.3 (4.1–4.5)	14.8 (14.4–15.1)	14.6 (13.8–15.4)
	Pulmonary diseases^†^	11,565	42.9 (36.2–49.7)	31.0 (30.3–31.7)	18.1 (17.7–18.5)	0.1 (0.1–0.1)	0.1 (0.0–0.1)
	Cardiovascular diseases^†^	1,555	5.1 (2.6–8.9)	3.0 (2.7–3.2)	0.8 (0.7–0.9)	1.5 (1.4–1.7)	1.9 (1.6–2.3)
	Neurology^†^	2,595	18.0 (13.1–23.7)	5.4 (5.1–5.8)	2.8 (2.6–2.9)	1.7 (1.6–1.8)	0.0 (0.0–0.1)
	Digestive diseases^†^	16,782	2.8 (1.0–5.9)	21.2 (20.6–21.8)	23.8 (23.3–24.2)	11.8 (11.5–12.1)	1.4 (1.1–1.7)
	Urology–nephrology^†^	2,876	0.0 (0.0–1.7)	3.9 (3.6–4.2)	3.1 (2.9–3.3)	2.8 (2.6–2.9)	0.1 (0.1–0.2)
	Gynecology^†^	190	0.0 (0.0–1.7)	0.2 (0.1–0.3)	0.3 (0.2–0.3)	0.2 (0.1–0.2)	0.1 (0.0–0.2)
	Dermatology^†^	8,098	1.4 (0.3–4.0)	2.2 (2.0–2.5)	3.1 (2.9–3.3)	12.3 (12–12.6)	20.6 (19.7–21.6)
	Endocrinology–metabolism disorders^†^	239	3.2 (1.3–6.5)	1.0 (0.8–1.1)	0.2 (0.1–0.2)	0.0 (0.0–0.0)	0.0 (0.0–0.1)
	Infectious diseases^†^	12,870	4.6 (2.2–8.3)	7.8 (7.4–8.2)	15.4 (15.0–15.8)	12.2 (11.9–12.6)	15.3 (14.5–16.1)
	Rheumatology–pain^†^	3,212	0.0 (0.0–1.7)	4.0 (3.7–4.3)	4.1 (3.9–4.3)	2.7 (2.6–2.9)	0.0 (0.0–0.0)
	Hematology^†^	141	0.0 (0.0–1.7)	0.7 (0.6–0.9)	0.1 (0.0–0.1)	0.0 (0.0–0.0)	0.0 (0.0–0.0)
	Poisoning^†^	146	1.4 (0.3–4.0)	0.4 (0.3–0.5)	0.2 (0.1–0.2)	0.1 (0.0–0.1)	0.1 (0.0–0.2)
	Others^†^	1,107	0.0 (0.0–1.7)	3.2 (2.9–3.5)	0.1 (0.0–0.1)	1.1 (1.0–1.2)	1.3 (1.1–1.6)
Surgical complaint	Head & neck trauma^†^	9,402	5.5 (2.9–9.5)	5.3 (5.0–5.7)	13.2 (12.8–13.6)	5.9 (5.7–6.1)	19.5 (18.6–20.4)
	Upper limb trauma^†^	10,032	0.5 (0.0–2.5)	4.3 (4.0–4.7)	6.3 (6.0–6.5)	15.0 (14.7–15.4)	12.0 (11.3–12.8)
	Lower limb trauma^†^	8,540	0.0 (0.0–1.7)	1.1 (0.9–1.2)	2.0 (1.8–2.1)	16.3 (15.9–16.6)	12.7 (12–13.5)
	Trunk–pelvis–urogenital trauma^†^	934	0.0 (0.0–1.7)	0.5 (0.4–0.6)	1.0 (0.9–1.2)	1.2 (1.1–1.3)	0.1 (0.1–0.2)
	Burns^†^	431	4.6 (2.2–8.3)	0.7 (0.6–0.8)	0.6 (0.5–0.7)	0.2 (0.2–0.3)	0.2 (0.1–0.3)
	Others^†^	591	8.3 (5–12.8)	1.3 (1.1–1.4)	0.8 (0.7–0.9)	0.2 (0.2–0.2)	0.0 (0.0–0.0)

*Values are presented as percentages with their 95% CI*.

† *p < 0.001 by level of triage*.

Finally, 60.9% (60.6–61.2) of the patients presented with at least one of the 11 most frequently encountered diagnoses in our study (Table 1). The most prevalent medical diagnoses were ENT infections and acute gastroenteritis with 15.9% (15.7–16.1) and 9.0% (8.8–9.1), respectively, while patients with upper and lower limb trauma were the most prevalent surgical cases with an overall prevalence of 18.4% (18.1–18.6).

### Severity Outcome

Overall, 94.0% of patients were discharged. The hospitalization rate significantly decreased with triage level, from 53.5% in Level 1 to 0.6% in Level 5 (*p* < 0.001). The ICU admission rates among patients defined as a “high-level emergency” (*pediaTRI* Levels 1 and 2) were significantly higher than those defined as “low-level.” PED LOS was also strongly associated with triage level: LOS decreased from 176 min in Level 1 to 96.6 min in Level 5 (*p* < 0.001). No deaths were reported in the PED during the study period.

### Validity

The high- and low-level emergencies assigned by *pediaTRI* were consistent with those defined by the PEWS score with an agreement of 84.5% (84.2–84.7) (Table 3). *pediaTRI* over-triaged 15,082 patients [15.0% (14.8–15.2), and under-triaged 528 (0.5% (0.5–0.6)].

**Table 3:** Agreement in triage between *pediaTRI* vs. PEWS in screening “high-level emergency” and “low-level emergency.”

**PEWS**	**Pediatric triage tool** ***pediaTRI***					
	**Stage 1^a^**	**Stage 2^a^**	**Stage 3^b^**	**Stage 4^b^**	**Stage 5^b^**	**Total**
≥4/9^a^	82^c^	1,628^c^	489^d^	38^d^	1^d^	2,238
≤ 3/9^b^	135^e^	14,947^e^	34,343^c^	41,264^c^	7,579^c^	98,268
Total	217	16,575	34,832	41,302	7,580	100,506

a *High-Level of emergency*.

b *Low-level of emergency*.

c *Agreement in triaging patients between pediaTRI vs. PEWS according to high-level emergencies*.

d *Under-triage of pediaTRI vs. PEWS*.

e *Over-triage of pediaTRI vs. PEWS*.

*pediaTRI* had a sensitivity of 76.4 (74.6–78.2) and a specificity of 84.7 (84.4–84.9) for identifying high-level emergencies (Table 4). The likelihood ratios were 5.0 (4.8–5.1) for high-level emergencies and 0.3 (0.3–0.3) for low-level emergencies. However, *pediaTRI* was more sensitive for infants under 3 months – 100.0% (66.4–100.0) for newborns (under 28 days of age) and 100.0% (75.3–100.0) for infants from 28 days to 3 months – but less specific at 14.8% (12.8–16.9) and 46.8% (44.8–48.8), respectively, for the same age groups. The negative predictive value was high in the overall sample (99.4) and their values remained high whatever the subgroup (from 90.5 to 100.0). The positive likelihood ratio values were mostly higher in the overall sample and in the subgroups than the negative likelihood ratios (0–32.9 vs. 0–1.1, respectively).

**Table 4:** Sensitivity, specificity, predictive values and likelihood ratios of *pediaTRI* vs. PEWS (Continued on next page).

			**High–Level emergency** % (CI95)*							
		***n***	***pediaTRI***	**PEWS**	**Sensitivity**	**Specificity**	**PPV**	**NPV**	**LR+**	**LR–**
Total^†^		100,506	16.7 (16.5–16.9)	2.2 (2.1–2.3)	76.4 (74.6–78.2)	84.7 (84.4–84.9)	10.2 (9.7–10.7)	99.4 (99.3–99.4)	5.0 (4.8–5.1)	0.3 (0.3–0.3)
Sex^†^	Male	55,166	18.1 (17.8–18.4)	2.3 (2.2–2.5)	78 (75.6–80.2)	83.3 (83.0–83.7)	10.0 (9.4–10.6)	99.4 (99.3–99.4)	4.7 (4.5–4.8)	0.3 (0.2–0.3)
	Female	45,333	15.0 (14.7–15.4)	2.1 (2.0–2.2)	74.3 (71.4–77.0)	86.2 (85.9–86.6)	10.4 (9.7–11.2)	99.4 (99.3–99.4)	5.4 (5.2–5.6)	0.3 (0.3–0.3)
Age	[0–28 d]	1,262	85.3 (83.3–87.2)	0.7 (0.3–1.3)	100.0 (66.4–100.0)	14.8 (12.8–16.9)	0.8 (0.4–1.6)	100.0 (98.0–100.0)	1.2 (1.1–1.2)	NA
	[28 d−3 m]^†^	2,462	53.5 (51.5–55.4)	0.5 (0.3–0.9)	100.0 (75.3–100.0)	46.8 (44.8–48.8)	1.0 (0.5–1.7)	100.0 (99.7–100.0)	1.9 (1.8–2.0)	NA
	[3 m−1 y]^†^	9,991	21.7 (20.9–22.5)	0.3 (0.2–0.5)	84.4 (67.2–94.7)	78.5 (77.7–79.3)	1.2 (0.8–1.8)	99.9 (99.9–100.0)	3.9 (3.4–4.6)	0.2 (0.1–0.4)
	[1 y−3 y]^†^	22,622	16.0 (15.5–16.5)	5.8 (5.5–6.1)	75.6 (73.2–77.9)	87.7 (87.2–88.1)	27.2 (25.8–28.7)	98.3 (98.1–98.5)	6.1 (5.8–6.4)	0.3 (0.3–0.3)
	[3 y−7 y]^†^	27,372	13.6 (13.2–14.0)	2.2 (2.0–2.4)	77.9 (74.4–81.1)	87.8 (87.4–88.2)	12.7 (11.6–13.8)	99.4 (99.3–99.5)	6.4 (6.1–6.7)	0.3 (0.2–0.3)
	[7 y−12 y]^†^	21,405	12.2 (11.8–12.6)	0.7 (0.6–0.8)	76.4 (68.6–83.1)	88.2 (87.8–88.7)	4.2 (3.5–5.1)	99.8 (99.7–99.9)	6.5 (5.9–7.2)	0.3 (0.2–0.4)
	[12 y−18 y]^†^	15,392	14.8 (14.2–15.3)	0.9 (0.7–1.0)	71.8 (63.2–79.3)	85.7 (85.1–86.3)	4.1 (3.4–5.0)	99.7 (99.6–99.8)	5.0 (4.5–5.6)	0.3 (0.3–0.4)
Type of complaint^†^	Medical	70,330	20.6 (20.3–20.9)	3.2 (3.0–3.3)	76.4 (74.6–78.1)	81.2 (80.9–81.5)	11.7 (11.2–12.2)	99.1 (99.0–99.1)	4.1 (4.0–4.2)	0.3 (0.3–0.3)
	Surgical	30,176	7.6 (7.3–7.9)	0.0 (0.0–0.1)	78.6 (49.2–95.3)	92.5 (92.2–92.8)	0.5 (0.2–0.9)	100.0 (100.0–100.0)	10.4 (7.9–13.7)	0.2 (0.1–0.6)
Medical complaint	ENT diseases^†^	9,200	5.4 (5.0–5.9)	0.2 (0.1–0.3)	41.2 (18.4–67.1)	94.6 (94.2–95.1)	1.4 (0.6–2.9)	99.9 (99.8–99.9)	7.7 (4.3–13.6)	0.6 (0.4–0.9)
	Pulmonary diseases^†^	11,565	45.2 (44.3–46.1)	17.2 (16.5–17.9)	78.6 (76.8–80.4)	61.8 (60.8–62.7)	29.9 (28.6–31.1)	93.3 (92.7–93.9)	2.1 (19.9–2.1)	0.3 (0.3–0.4)
	Cardiovascular diseases	1,555	32.2 (29.8–34.5)	3.3 (2.5–4.3)	31.4 (19.1–45.9)	67.8 (65.4–70.2)	3.2 (1.8–5.1)	96.7 (95.4–97.7)	1.0 (0.6–1.5)	1.0 (0.8–1.2)
	Neurology^†^	2,595	36.1 (34.2–38.0)	2.0 (1.5–2.6)	96.1 (86.5–99.5)	65.1 (63.2–67.0)	5.2 (3.9–6.9)	99.9 (99.6–100.0)	2.8 (2.6–3.0)	0.1 (0.0–0.2)
	Digestive diseases^†^	16,782	21.0 (20.4–21.6)	0.1 (0.1–0.2)	82.6 (61.2–95.0)	79.1 (78.5–79.7)	0.5 (0.3–0.8)	100.0 (99.9–100.0)	4.0 (3.3–4.8)	0.2 (0.1–0.5)
	Urology–nephrology	2,876	22.4 (20.8–23.9)	0.0 (0.0–0.1)	NA	77.6 (76.1–79.2)	0.0 (0.0–0.6)	100.0 (99.8–100.0)	NA	NA
	Gynecology	190	15.3 (10.5–21.2)	0.0 (0.0–1.9)	NA	84.7 (78.8–89.5)	0.0 (0.0–11.9)	100.0 (97.7–100.0)	NA	NA
	Dermatology	8,098	4.6 (4.2–5.1)	0.1 (0.0–0.2)	42.9 (9.9–81.6)	95.4 (94.9–95.9)	0.8 (0.2–2.3)	99.9 (99.9–100.0)	9.3 (4.0–22.1)	0.6 (0.3–1.1)
	Endocrino–metabo. disorders	239	69.5 (63.2–75.2)	2.1 (0.7–4.8)	100 (47.8–100.0)	31.2 (25.3–37.6)	3.0 (1.0–6.9)	100.0 (95.1–100.0)	1.5 (1.3–1.6)	0 (0–NA)
	Infectious diseases^†^	12,870	10.1 (9.6–10.6)	0.6 (0.4–0.7)	42.5 (31.0–54.6)	90.1 (89.6–90.6)	2.4 (1.6–3.4)	99.6 (99.5–99.7)	4.3 (3.3–5.6)	0.6 (0.5–0.8)
	Rheumatology–pain	3,212	20.6 (19.2–22.0)	0.2 (0.1–0.4)	50.0 (11.8–88.2)	79.5 (78.0–80.9)	0.5 (0.1–1.3)	99.9 (99.7–100.0)	2.4 (1.1–5.4)	0.6 (0.3–1.4)
	Hematology	141	83.7 (76.5–89.4)	1.4 (0.2–5.0)	100.0 (15.8–100.0)	16.5 (10.8–23.8)	1.7 (0.2–6.0)	100.0 (85.2–100.0)	1.2 (1.1–1.3)	0 (0–NA)
	Poisoning	146	44.5 (36.3–53.0)	0.0 (0.0–2.5)	NA	55.5 (47.0–63.7)	0.0 (0.0–5.5)	100.0 (95.5–100.0)	NA	NA
	Others	1,107	47.9 (44.9–50.9)	0.0 (0.0–0.3)	NA	52.1 (49.1–55.1)	0.0 (0.0–0.7)	100.0 (99.4–100.0)	NA	NA
Surgical complaint	Head & neck trauma^†^	9,402	9.5 (8.9–10.1)	0.1 (0.0–0.1)	100.0 (54.1–100.0)	90.6 (90.0–91.2)	0.7 (0.2–1.5)	100.0 (100.0–100.0)	10.6 (10.0–11.3)	0 (0–NA)
	Upper limb trauma	10,032	7.2 (6.7–7.7)	0.0 (0.0–0.1)	0.0 (0.0–84.2)	92.8 (92.3–93.3)	0.0 (0.0–0.5)	100.0 (99.9–100.0)	0 (0–NA)	1.1 (1.1–1.1)
	Lower limb trauma	8,540	2.0 (1.8–2.4)	0.0 (0.0–0.1)	66.7 (9.4–99.2)	98.0 (97.7–98.3)	1.1 (0.1–4.1)	100.0 (99.9–100.0)	32.9 (14.6–74.2)	0.3 (0.1–1.7)
	Trunk-pelvis-urogen. trauma	934	8.9 (7.1–10.9)	0.0 (0.0–0.4)	NA (0–100.0)	91.1 (89.1–92.9)	0.0 (0.0–4.3)	100.0 (99.6–100.0)	NA	NA
	Burns	431	28.5 (24.3–33.1)	0.2 (0.0–1.3)	100.0 (2.5–100.0)	71.6 (67.1–75.8)	0.8 (0.0–4.4)	100.0 (98.8–100.0)	3.5 (3.0–4.1)	0 (0–NA)
	Others	591	38.6 (34.6–42.6)	1.0 (0.4–2.2)	83.3 (35.9–99.6)	61.9 (57.8–65.8)	2.2 (0.7–5.0)	99.7 (98.5–100.0)	2.2 (1.5–3.2)	0.3 (0.0–1.6)
Diagnosis	ENT infectious diseases^†^	15,978	7.1 (6.7–7.5)	1.6 (1.4–1.8)	41.8 (35.7–48.0)	93.5 (93.1–93.9)	9.6 (8.0–11.5)	99.0 (98.8–99.1)	6.4 (5.5–7.5)	0.6 (0.6–0.7)
	Acute gastroenteritis^†^	9,002	22.2 (21.3–23.0)	0.3 (0.2–0.5)	56.7 (37.4–74.5)	77.9 (77.1–78.8)	0.9 (0.5–1.4)	99.8 (99.7–99.9)	2.6 (1.9–3.5)	0.6 (0.4–0.8)
	Asthma^†^	2,856	59.9 (58.1–61.7)	35.9 (34.1–37.6)	89.4 (87.3–91.2)	56.6 (54.2–58.8)	53.5 (51.1–55.9)	90.5 (88.6–92.1)	2.1 (1.9–2.2)	0.2 (0.2–0.2)
	Bronchiolitis^†^	1,654	66.8 (64.5–69.1)	12.8 (11.2–14.5)	84.9 (79.4–89.4)	35.9 (33.4–38.4)	16.3 (14.2–18.6)	94.2 (91.9–96.0)	1.3 (1.2–1.4)	0.4 (0.3–0.6)
	Flu^†^	1,669	15.9 (14.2–17.8)	3.1 (2.3–4.1)	53.8 (39.5–67.8)	85.3 (83.5–87.0)	10.5 (7.1–14.9)	98.3 (97.5–98.9)	3.7 (2.8–4.8)	0.5 (0.4–0.7)
	Fever^†^	4,605	16.2 (15.1–17.3)	2.2 (1.8–2.7)	56.3 (46.2–66.1)	84.7 (83.6–85.8)	7.8 (6.0–9.9)	98.8 (98.4–99.1)	3.7 (3.1–4.4)	0.5 (0.4–0.6)
	Mild trauma brain injury^†^	5,400	10.6 (9.8–11.5)	0.1 (0.1–0.3)	100.0 (59.0–100.0)	89.5 (88.7–90.3)	1.2 (0.5–2.5)	100.0 (99.9–100.0)	9.5 (8.8–10.3)	NA
	Upper limb trauma	10,062	8.0 (7.5–8.6)	0.0 (0.0–0.1)	0.0 (0.0–84.2)	92.0 (91.4–92.5)	0.0 (0.0–0.5)	100.0 (99.9–100.0)	NA	1.1 (1.1–1.1)
	Lower limb trauma^†^	8,402	3.0 (2.7–3.4)	0.0 (0.0–0.1)	100.0 (15.8–100.0)	97.0 (96.6–97.3)	0.8 (0.1–2.8)	100.0 (100.0–100.0)	33.1 (29.3–3.7)	NA
	Burn	461	26.7 (22.7–31.0)	0.0 (0.0–0.8)	NaN (0.0–100.0)	73.3 (69.0–77.3)	0.0 (0.0–3.0)	100.0 (98.9–100.0)	NA	NA
	Visceral pathologies	1,142	40.6 (37.8–43.5)	0.4 (0.1–0.9)	50.0 (6.8–93.2)	59.4 (56.5–62.3)	0.4 (0.1–1.5)	99.7 (98.9–100.0)	1.2 (0.5–3.3)	0.8 (0.3–2.2)

* *Immediate and very urgent category; LR+=likelihood ratio for high-level emergency triage test result; LR–=likelihood ratio for low-level emergency triage test result*.

*Sensitivity % (CI95) = high-level emergency; Specificity % (CI95) = low-level emergency*;

† *p < 0.001 per type of level of emergency assigned by pediaTRI vs PEWS; NA Not applicable*.

The performance values differed according to the category of chief complaint. Specificity values for medical complaints, were moderate for pulmonary, cardiovascular system, neurology, digestive, urology-nephrology, and rheumatology conditions, and low for endocrinology-metabolism disorders, hematology, and poisoning. Specificity values for surgical complaints were mainly high contrasting with a lower sensitivity except for head and neck trauma and burns. The most frequent medical reason assessed by PEWS as a high-level emergency was pulmonary related (17.2%; 16.5–17.9), followed by cardiovascular disease (3.3%; 2.5–4.3), endocrinology-metabolism disorders (2.1%; 0.7–4.8), and finally neurology (2.0%; 1.5–2.6). The corresponding assessments by *pediaTRI* were 45.2% (44.3–46.1), 32.2% (29.8–34.5), 69.5% (63.2–75.2) and 36.1% (34.2–38.0), respectively. High-level emergency cases for surgical reasons ranged from 0.0% (0.0–0.1) to 1.0% (0.4–2.2) for PEWS and from 2.0% (1.8–2.4) to 38.6% (34.6–42.6) for *pediaTRI*.

For the main medical diagnoses, *pediaTRI* had higher specificity (73.3–97.0) than sensitivity (41.8–56.7) for ENT infectious diseases, acute gastroenteritis, Flu' and unknown fever. Sensitivity was higher than specificity for asthma and bronchiolitis (89.4 and 84.9 vs. 56.6 and 35.9, respectively). For surgical diagnoses, as few patients were classified by PEWS as high-level emergency, values of specificity varied from 59.4 to 97.0 while those of sensitivity were not systematically interpretable.

### Validation Cohort

The patient characteristics per triage level were similar between the periods 2016–17 and 2018–19. Although LOS and the level of emergency followed the same downward trends, we reported significantly longer PED LOS during 2018–19 with 205 min, 146 min, 134.6 min, 118.6 min, and 107.2 min respectively from Level 1 to 5. Prevalence of high- and low-level emergencies between *pediaTRI* and PEWS between the two periods were also similar with an agreement of 84.9% (84.7–85.1), an over-triage of 14,522 patients [14.5% (14.3–14.8)], and an under-triage 528 patients (0.6% (0.6–0.7). We reported similar values of performance of *pediaTRI* between the two periods except for lower sensitivities for surgical reasons (46.2 vs.78.6%) and for digestive diseases (60.7 vs. 82.6) during the 2018–19 period.

## Discussion

### Principal Findings and Interpretation

The *pediaTRI* triage tool has an overall moderate validity compared to the PEWS system with a sensitivity of 76.4% and specificity of 84.7%. The agreement with PEWS was 84.5%, with over-triage in 15.0% of the patients and under-triage in 0.5% (mostly in one category).

However, the performance of *pediaTRI* varies according to the patient profile. In children under 3 months, sensitivity was high (100% for those under 28 days and 100% for 1 to 3 months) contrasting with a poor specificity (respectively, 14.8 and 46.8%) due to potential “risky” situations in this young population. The sensitivity for children with a medical history or existing chronic disease (for example, a cardiovascular, neurological, endocrinological, or hematological condition) was much higher than the specificity. For these particular cases, over-triage is necessary whatever the chief complaint so that these children do not wait too long before being seen by a physician as their clinical condition could deteriorate rapidly.

The performance of *pediaTRI* also varies according to the subcategory of the chief complaint. Each subcategory contains many items, some of which are prevalent – such as fever (*n* = 11,827), and vomiting (*n* = 6,685) – whereas these items are not accurate enough to define a severity level. Thus, it would be necessary to do more in-depth analysis to assess the performance of each item and evaluate its relevance, or to reclassify or delete insufficiently relevant items.

Severity outcomes (ICU admission, hospitalization, PED LOS) correlated inversely with the *pediaTRI* triage level: the severity profile decreased from Level 1 to 5.

The variability of the performance values of *pediaTRI* underlines the need to provide a framework to mitigate the risk of failing to identify critically ill patients. Consequently, we compiled a guide to ensure that patients are triaged safely. Firstly, the duration of the triage should be long enough to assess the patient adequately without unnecessarily delaying access to care. We recommend that the triage should initially focus on the recognition and management of critically ill children based on the ABCDE approach of the Pediatric Life Support of the European Resuscitation Council Guidelines (30). In the absence of a life-threatening condition, the trained nurse then performs a structured clinical interview to detect the presence or absence of signs system by system (i.e., cardiovascular, pulmonary,..) to capture any significant signs that could upgrade the triage level. The computerized version of the *pediaTRI* tool includes pop-up messages to inform the nurse if essential data (vital signs) are missing for the triage process. The nurse can upgrade the triage level if they disagree with the one suggested by *pediaTRI*, but not downgrade the level. Finally, the nurses are encouraged to ask the on-call pediatrician to confirm the final triage level of a patient at any time if in doubt.

### Comparison With Other Studies

Most triage tool validation studies are based on correlations with resource utilization rather than comparing performance against a gold standard (6) (Appendix 4), mainly the Manchester Triage Scale (MTS). Our performance values seem better than those of the Australasian Triage Scale, the Canadian Emergency Department Triage and Acuity Scale, the Emergency Severity Index (5), and similar to those of MTS (31–33) with sensitivity values ranging from 57 to 83% and specificity values from 69 to 93.7% depending on the study. However, the prevalence of *pediaTRI* for over-triage and under-triage appears to be better than those estimated for MTS. Indeed, while 84.5% of the patients in our study were correctly triaged, 15% were over-triaged and only 0.5% under-triaged.

Except for patients under 3 months old, for whom *pediaTRI* was more sensitive than MTS (100% vs. 50 – 79%) due to the over-triage setting by default as explained previously, the performance values of *pediaTRI* are in the range of those reported in the literature (32, 34): i.e., with a higher specificity (pediaTRI: 78.5 – 88.2% vs. others: 69 – 88%) than sensitivity (*pediaTRI*: 75.6 – 84.4% vs. others: 65 – 67%). Unfortunately, we are not able to interpret our results concerning the performance of *pediaTRI* by subcategory of chief complaint or specific diagnosis in relation to other works, as these outcomes are missing in the literature.

However, our reference standard differed from previous studies that used a benchmark triage tool for identifying high-level emergencies. The benchmarks were mainly created in situ by a committee of experts and differed from one study to another due to different practices. Although the performance of our reference standard (PEWS) may not be perfect, it has nonetheless been shown that a high PEWS score is correlated with a higher risk of patient morbidity and mortality that defines high-level emergency (16, 18, 19, 28, 35). Nevertheless, we also report a strong association between triage level and markers of severity, namely hospitalization rates and PED LOS. As reported in previous studies, hospitalization (especially ICU admission) and a longer PED LOS are associated with high-level emergencies. Our hospitalization rates are in the lower range of those reported in the literature (11, 13, 31, 36–40) with 53.5–100%, 28.6–46%, 8–17%, 1.2–6%, and 0–2%, respectively, for triage levels 1 to 5. Similarly, our PED LOS are also slightly lower than those reported in the literature (11, 13, 36, 37, 40, 41) with 102–334 min, 221–309 min, 191–262 min, 96 −186 min, and 66–160 min, respectively, for triage levels 1 to 5: as expected, high-acuity patients present conditions that require more procedures, time for stabilization, and work-up. Thus, hospitalization rates and PED LOS would appear to be useful markers to validate triage tools.

### Strengths and Limitations of the Study

#### A Large-Scale Population Study

Our study analyzed data from a large number of patients (> 1,00,000) over 2 successive years (2016–2017), thereby avoiding any bias related to temporality (season, week, day, time of day/night) and suggesting that our results are representative of all the pathologies encountered in a large-scale PED in France. Statistical analyses carried out on a large control cohort (> 100,000 patients from 2018–2019) served as validation for the sample cohort.

The prevalence of our five severity levels were in line with those reported in previous studies, with prevalence ranging from 0.3–16.5%, 4.5–26.4%, 20.0–55.0% 17.2–52.1%, and 0.1–21.2% for Levels 1 to 5, respectively (13, 34, 41, 42) (Appendix 3).

The prevalence of medical diagnoses in our study were also similar to that of previous studies, from 1.1–5.4% for bronchiolitis (43, 44), 2.5–16.1% for asthma (36, 44), and 4.9–6.4% for acute gastroenteritis (42). However, we had more cases of limb and head trauma compared with other studies (11, 42, 43). Limb trauma in our study comprised all types of diagnosis, such as contusion and sprain, wound, and dislocation which explains our higher prevalence compared to Gravel et al. (36) and Acworth et al. (42). Likewise, head injuries in our study were more frequent compared with the study by Christoffel et al. (43) because our definition of head injuries included mild contusion with or without scalp wound as well as severe head injuries (including skull bone fracture and post-traumatic intracranial hemorrhage) (Appendix 5).

#### A Tool Specifically Used for Triaging Pediatric Patients

In the absence of a standardized pediatric triage tool in France, our *pediaTRI* tool shares the characteristics of the other large-scale tools: it is a computerized tool with five levels of severity that analyzes symptoms and vital signs. However, *pediaTRI* was specifically designed for evaluating pediatric patients while the existing pediatric triage tools are adaptations of tools designed for adult patients. Finally, *pediaTRI* can be used by any trained nurse without specific clinical skills.

#### The Reference Standard

PEWS can be applied to any PED to identify high-level emergencies. It is a simple and objective tool that scores the severity of patients based on three parameters: the neurologic, cardiovascular, and respiratory systems. No lab workup or complementary tests are required which means that the nurse can quickly triage their pediatric patients. The PEWS has been validated with good reproducibility (18, 35, 45–47) due to its simplicity, and allows both initial assessment and continuous monitoring irrespective of the hospital unit. Although it was designed to detect changes over time, its implementation in EDs to detect early clinical deterioration of patients has been studied with a score that changes little over time as the patients are mostly in the ED for a relatively limited time. (19, 27, 45). Thus, a high PEWS score (≥4/9), defined as a predictor of ICU admission, assigned in a PED setting can be an indicator of high-level emergency. However, authors agree that PEWS alone is insufficient for assessing or triaging patients.

There are some other limitations to our study. First, this was a single-center study which somewhat limits the generalizability of our results despite the large sample size.

Furthermore, data were missing for around 6 % of the children, especially for patients with unreliable vital signs (2%). These were considered as abnormal or alarming because of difficult conditions during the measurement as the children were crying or scared. The PED environment is not the ideal setting to record reliable vital signs (48) which is why we considered the vital signs as normal by default. Although we included a large patient sample, we observed a very low rate of Level 1 cases (*n* = 217, 0.2%). Level 1 (immediate emergency requiring resuscitation and emergency care) is rare in pediatrics and our prevalence is in the lower range of those in the literature (from 0.3 to 16.5%) (Appendix 3). Differences in the prevalence of such cases may be partly explained by the organization of the healthcare system according to the country. In France, mobile emergency and intensive care service units (Service Mobile d'urgence et de Réanimation-SMUR) (49, 50) are triggered before hospital involvement to manage patients in a critical condition and these patients are admitted directly in the ICU without being assessed in the PED.

We compared our 5-level *pediaTRI* tool with PEWS, a reference standard based on identifying patients at risk of clinical deterioration by classifying them into two groups: high-level vs. low-level emergencies. However, the PEWS screens for signs of severity and does not include analysis of the chief complaint suggesting that it might not be as efficient as *pediaTRI* for analyzing medical vs. surgical complaints. It would be interesting to use a modified PEWS that integrates pain and disability, for example. However, we considered that PEWS was an appropriate reference standard because of its practicability (standardized evaluation), and feasibility (using a simple score for triaging patients) even though its validity has been challenged in the literature (51). Furthermore, Seiger et al. found that the ability of the PEWS for predicting hospitalization was low to moderate (16), but moderate to good for ICU admission. In a prospective study, Breslin et al. (45) demonstrated an area under the curve of 0.68 to detect the need for hospitalization (but 0.80 in the subpopulation of respiratory pathologies). Mandell et al. (52) concluded that no threshold was of sufficient sensitivity or specificity to be clinically useful for identifying with certainty the need for ICU admission, although a high PEWS was statistically associated with an increased risk of admission to intensive care. However, setting the threshold too high would result in failure to identify some seriously ill children, while setting it too low would unnecessarily utilize precious time and resources. In our study, we defined a PEWS ≥ 4 (26–28) as denoting a high-level of emergency as this threshold is significantly related to ICU admission. Finally, the performance of *pediaTRI* was compared with PEWS as a 2-level pediatric triage tool to screen for high- and low-level emergencies rather than a 5-level tool as recommended in the literature (5, 31, 32, 34, 53, 54). Meanwhile, our results based on the markers of severity (ICU admission, hospitalization, PED LOS) are in agreement with those reported in the literature.

## Conclusion

Our *pediaTRI* tool used by trained nurses showed a moderate to good validity to screen high-level emergencies in pediatric emergency care but its performance differs according to the subcategory of the chief complaint and the patient profile. Multicenter studies are needed to assess its validity and reliability as a 5-level triage tool. Experienced nurses with clinical skills at the triage zone could improve the triage process and, in the future, we could aim to introduce the “clinical nurse” status in France.

## Strengths and Limitations of This Study

- This is the first study to validate a French pediatric triage tool, *pediaTRI*, compared with a reference standard.
- We analyzed a large study sample over 2 successive years in the 4^th^ largest 24/7 PED in France.
- Statistical analyses carried out on a large control cohort (>100,00 patients from 2018–2019) served as validation for the sample cohort.
- The performance of *pediaTRI* was comprehensively assessed according to the age of the patients, the type of complaint (medical vs. surgical), and the category of complaint.
- This was a single-center study limiting its applicability in emergency departments across France.
- The PEWS, used as the reference standard, did not allow effective comparison of performance of *pediaTRI* with previous studies.

## Data Availability Statement

The raw data supporting the conclusions of this article will be made available by the authors, without undue reservation.

## Ethics Statement

Ethical review and approval was not required for the study on human participants in accordance with the local legislation and institutional requirements. Written informed consent from the participants' legal guardian/next of kin was not required to participate in this study in accordance with the national legislation and the institutional requirements. Verbal informed consent was provided.

## Author Contributions

AT was responsible for protocol development, analyses, writing of the paper and the main author of the paper. PV supervised the protocol development, supervised the analyses, writing of the paper and had a main contribution in editing the paper. CR supervised the protocol development, data collection, and writing of the paper. ER was responsible for data collection. EF, EB, and OH supervised the writing of the paper. HH supervised the protocol development, and writing of the paper. CP and SG supervised the protocol development, analyses, and writing of the paper. All the authors contributed to the article and approved the submitted version.

## Conflict of Interest

The authors declare that the research was conducted in the absence of any commercial or financial relationships that could be construed as a potential conflict of interest.

## Publisher's Note

All claims expressed in this article are solely those of the authors and do not necessarily represent those of their affiliated organizations, or those of the publisher, the editors and the reviewers. Any product that may be evaluated in this article, or claim that may be made by its manufacturer, is not guaranteed or endorsed by the publisher.
