# Supplementary material for: Validation of the Computerized Pediatric Triage Tool, pediaTRI, in the Pediatric Emergency Department of Lenval Children's Hospital in Nice: A Cross-Sectional Observational Study
Source: Front Pediatr. 2022 Apr 26;10:840181. doi: 10.3389/fped.2022.840181 (PMC9113392; doi:10.3389/fped.2022.840181)
Supplement: Supplementary file 1 [file Data_Sheet_1.pdf]

Patient ID

Identity of the nurse: Date : |__|__|/|__|__|/|__|__|__|__| Time: |__|__|: |__|__|

Glasgow(/15):|__|__|Temp° C:|__|__|,|__| Weight(kg):|__|__|,|__| BP(mmHg):|__|__|__|/|__|__|__| HR(/mn):|__|__|__| RR(/mn):|__|__|__|SaO2(%):|__|__|Pain score:|__|__|

Psychological/psychiatric conditions: Level 2
1a❒ sexual assault
1b❒ suspicion of child abuse
1c❒ restless and/or aggressive child

Skin coloration :
Level 2
2a❒ intense pallor
2b❒ cyanosis
2c❒ marbling extremities
2d❒ grayish skin
Level 3
2e❒ moderate pallor
2f❒ jaundice

Pain scale :
EVENDOL (<7y)
3a ❒ > 8: level 2
3b ❒ between 4 & 8: level 3
3c ❒< 4 : level 4
3g ❒ **testicular pain** level 2
VAS (≥ 7y)
3d❒ > 5 : level 2
3e❒ between 3 & 5: level 3
3f❒ < 3: level 4

Risky or specific conditions : Level 2
4a❒ newborn (<28d)
4b❒ sickle cell disease
4c❒ immunosuppression
4d❒ severe allergy
4e❒ blood diseases

Fever :
Level 2
5a❒ < 3 months old
5b❒ shivering, marbling
5c❒ whining
5d❒ seizure
5e❒ purpura or petechiae
5f ❒ > 40.5° Celsius

Level 3
5g❒ < 6 months old
5h❒ deterioration of general condition

Level 4
5i ❒ < 2 years old
5j❒ fever > 48h

Respiratory system :
Level 2
6a ❒ < 3 months old
6b ❒ apnea
6c ❒ expiratory whine
6d ❒ nasal flaring
6e ❒ speech difficulty
6f ❒ sweating
6g ❒ obstructed chest
6h ❒ refusal to feed or shortness of breath during feeding

Level 3
6i ❒ hoarse cough
6j ❒ accelerated breathing, no sign of major struggle
6k ❒ moderate seesaw pattern of breathing

Level 4
6l ❒ discrete signs of struggle (slight pull, slight swaying) with usual physical activity (playing, feeding)

Neurologic system
Level 2
7a❒ Glasgow score < 15
7b❒ abnormal movements
7c❒ neck stiffness
7d❒ visual disturbances (double vision, photophobia)
7e❒ severe paroxysmal headache
7f ❒ headache with repeated vomiting

Level 3
7g❒ seizure lasting < 1 hour with return to a normal neurological state
7h❒ persistent severe headache without vomiting

Level 4
7i❒ headache with normal physical activity (play)
7j❒ convulsion lasting several hours for a known epilepsy disorder

Digestive system :
Level 2
8a❒ sunken eyes
8b❒ skin fold
8c❒ dry mucous membranes
8d❒ bloody, bilious or fecal vomiting
8e❒ total food intolerance
8f❒ gas and fecal matter obstruction
8g❒ rectal bleeding
8h❒ abdominal pain with discoloration or fainting

Level 3
8i❒ repeated diarrhea or vomiting with oral rehydration possible
8j❒ abdominal pain without discoloration or fainting
8k❒ mucous and / or bloody diarrhea

Level 4
8l❒ isolated vomiting with normal feeding
8m❒ isolated diarrhea with normal general condition
8n❒ digestive disorder with normal feeding

Head trauma :
Level 1
9a❒Glasgow score < 14

Level 2
9b❒ prolonged initial loss of consciousness (> 5 mn)
9c❒ delayed initial loss of consciousness
9d❒ >3 vomiting
9e❒ neurological symptoms (drowsiness, restlessness, pupillary asymmetry)

Level 3
9f❒ with initial loss of consciousness < 5 mn

Level 4
9g ❒ with short initial loss of consciousness < 1 mn

Limb trauma
Level 1
10a❒ active bleeding

Level 2
10b❒ limb deformation
10c❒ motor or sensory neurological deficit
10d❒ reduction of the distal pulse in the injured limb
10e❒ under plaster: abnormal coloration, pain or motor or sensory deficit

Level 3
10f❒ functional impotence without limb deformation

Level 4
10g❒ No functional impotence, no limb deformation

Burn :
Level 1
11a❒ location: face, neck, perineum, thorax
11b❒ any extensive burn > 10% body (1% = 1 child's hand)

Level 2
11c❒ burns due to fire, corrosives, chemicals, electrical
11d❒ < 1 year old
11e❒ fever

Level 3
11f❒ simple blister
11g❒ burn < 1% body

Wound: Level 2
12a❒ active and pulsatile bleeding
12b❒ active and persistant bleeding with compression
12c❒ location: eye, face, neck, thorax, abdomen
12d❒ crushing mechanism
12e❒ quasi-circumferential section (limb, finger)

Poisoning : Level 2
13a❒ voluntary, teenager
13b❒ cardiovascular, respiratory, neurological risks
13d❒ mushroom
13f❒ known toxicity: acetaminophene, aspirine, alcohol, gasoline, carbon monoxide, corrosive products

Level of triage:
Level 1 : life-threatening emergency (cardiac arrest, heart/respiratory/neurological failure)
Level 2 : very urgent, fractile response : 0-20 mn
Level 3 : urgent, fractile response : 0-60 mn
Level 4 : consultation, fractile response : 0-120 mn
Level 5 : non urgent, fractile response : 0-240 mn
