# Supplementary material for: Validation of the Computerized Pediatric Triage Tool, pediaTRI, in the Pediatric Emergency Department of Lenval Children's Hospital in Nice: A Cross-Sectional Observational Study
Source: Front Pediatr. 2022 Apr 26;10:840181. doi: 10.3389/fped.2022.840181 (PMC9113392; doi:10.3389/fped.2022.840181)
Supplement: Supplementary file 2 [file Data_Sheet_2.pdf]

Appendix 2. statistical analysis carried out on the validation cohort

Table A1. Characteristics of the sample by level of triage

		n	**Level 1** (n=217)	**Level 2** (n=16003)	**Level 3** (n=34623)	**Level 4** (n=41335)	**Level 5** (n=8076)
**Total†**		100,254	0.2 (0.2-0.2)	16.0 (15.7-16.2)	34.5 (34.2-34.8)	41.2 (40.9-41.5)	8.1 (7.9-8.2)
**Sex†**	Male	54,993	61.3 (54.5-67.8)	58.7 (57.9-59.5)	55.7 (55.2-56.2)	52.9 (52.4-53.4)	53.5 (52.4-54.6)
	Female	45,249	38.7 (32.2-45.5)	41.3 (40.5-42.1)	44.3 (43.8-44.8)	47.1 (46.6-47.6)	46.5 (45.4-47.6) ‡
**Age†**	[0-28d[	1,264	3.2 (1.3-6.5)	7.4 (7.0-7.9) ‡	0.0 (0.0-0.1) ‡	0.1 (0.1-0.1)	0.1 (0.0-0.2)
	[28d-3m[	2,630	6.0 (3.2-10.0)	10.3 (9.9-10.8) ‡	1.4 (1.3-1.6)	0.9 (0.8-1.0) ‡	1.1 (0.9-1.3)
	[3m-1y[	9,800	14.7 (10.3-20.2)	10.6 (10.1-11.1) ‡	11.6 (11.3-12.0)	7.2 (6.9-7.4)	13.2 (12.5-14.0) ‡
	[1y-3y[	22,319	20.7 (15.5-26.7)	20.1 (19.4-20.7) ‡	24.9 (24.4-25.3)	20.7 (20.3-21.1)	23.6 (22.6-24.5)
	[3y-7y[	26,159	24.0 (18.4-30.2)	20.5 (19.9-21.1)	28.5 (28.0-28.9)	26.3 (25.9-26.7)	25.9 (25.0-26.9)
	[7y-12y[	21,392	11.1 (7.2-16.0)	15.5 (15.0-16.1)	18.7 (18.3-19.2)	25.9 (25.5-26.3)	21 (20.1-21.9)
	[12y-18y[	16,690	20.3 (15.1-26.2)	15.5 (15.0-16.1)	14.8 (14.4-15.2)	18.9 (18.5-19.3)	15.1 (14.4-15.9)
**Outcome†**	Intensive care unit	121	7.8 (4.6-12.2)	0.5 (0.4-0.6)	0.0 (0.0-0.1)	0.0 (0.0-0.0)	0.0 (0.0-0.0)
	Hospitalization[a]	5,687	56.2 (49.3-62.9)	19.9 (19.3-20.5) ‡	5.4 (5.2-5.7) ‡	1.1 (1.0-1.2)	0.5 (0.4-0.7)
	Transfer[b]	122	0.5 (0.0-2.5)	0.5 (0.4-0.6)	0.1 (0.1-0.1)	0.0 (0.0-0.1)	0.0 (0.0-0.1)
	Discharged	94,324	35.5 (29.1-42.2)	79.1 (78.5-79.8)	94.5 (94.2-94.7)	98.8 (98.7-98.9)	99.4 (99.3-99.6) ‡
**Diagnosis**	ENT infectious diseases[M]†	15,108	0.0 (0.0-1.7)	6.2 (5.8-6.6)	16.0 (15.6-16.4) ‡	17.2 (16.8-17.6) ‡	18.1 (17.3-19.0) ‡
	Acute gastroenteritis[M]†	8,502	1.8 (0.5-4.7)	10.3 (9.9-10.8) ‡	13.9 (13.6-14.3)	4.6 (4.4-4.8)	1.5 (1.3-1.8)
	Asthma[M]†	3,073	17.5 (12.7-23.2)	10.5 (10.0-11.0)	3.6 (3.4-3.8)	0.2 (0.2-0.2)	0.3 (0.2-0.4)
	Bronchiolitis[M]†	1,682	9.2 (5.7-13.9)	6.3 (5.9-6.6)	1.7 (1.5-1.8)	0.2 (0.1-0.2)	0.2 (0.1-0.3)
	Flu[M]†	1,080	0.0 (0.0-1.7)	1.0 (0.9-1.2)	1.4 (1.3-1.5)	0.9 (0.8-1.0)	0.8 (0.6-1.0)
	Fever[Mc]†	5,044	2.8 (1.0-5.9)	4.5 (4.2-4.8)	6.5 (6.2-6.7)	4.3 (4.1-4.5)	3.7 (3.3-4.1)
	Mild trauma brain injury[S]†	5,238	5.5 (2.9-9.5)	3.3 (3.0-3.6)	6.8 (6.5-7.1)	2.7 (2.6-2.9) ‡	15.2 (14.4-16.0)
	Upper limb trauma[S]†	9,702	2.3 (0.8-5.3)	4.3 (4.0-4.6)	6.7 (6.5-7.0)	14 (13.7-14.3)	11.1 (10.5-11.8)
	Lower limb trauma[S]†	8,171	0.9 (0.1-3.3)	1.4 (1.3-1.6)	2.6 (2.4-2.8)	15.2 (14.8-15.5)	9.6 (9.0-10.3)
	Burn[S]†	453	2.3 (0.8-5.3)	0.6 (0.5-0.7)	0.5 (0.4-0.6)	0.4 (0.3-0.4)	0.2 (0.1-0.3)
	Visceral pathologies[Sd]†	1,297	0.5 (0.0-2.5)	3.4 (3.1-3.7)	1.6 (1.5-1.8)	0.4 (0.4-0.5)	0.1 (0.1-0.2)
**Length of stay (mn)[e]†**			205.2 ± 103.1	146.0 ± 96.1 ‡	134.6 ± 85.2 ‡	118.6 ± 74.8 ‡	107.2 ± 71.7 ‡

Values are presented as percentages with their 95% CI or means (Length of stay) with their standard deviation

†p<0.001 by level of triage; M: Top 6 of the medical diagnosis, S: Top 5 of the surgical diagnosis;

‡p<0.001 by periods: 2016-2017 versus 2018-2019

[a] Medical and surgical units

[b] 122 children were transferred to 2 pediatric units (hematology, neonatology) that are located at the Archet university hospital of Nice (2miles away from the Lenval Children's hospital)

[c] Fever of unknown origin

[d] Appendicitis, occlusive pathologies, hernial pathologies, ovarian pathologies

[e] Length of stay defined as the duration between the time of the admission and the time of the exit of the PED (in minutes)

Table A2. Characteristics of complaints by level of triage

		n	**Level 1** (n=217)	**Level 2** (n=16003)	**Level 3** (n=34623)	**Level 4** (n=41335)	**Level 5** (n=8076)
**Type of complaint**†‡	Medical	70,909	84.3 (78.8-88.9)	88.0 (87.5-88.5)	75.3 (74.9-75.8)	63.0 (62.5-63.4)	56.3 (55.2-57.4)
	Surgical	29,345	15.7 (11.1-21.2)	12.0 (11.5-12.5) ‡	24.7 (24.2-25.1)	37.0 (36.6-37.5)	43.7 (42.6-44.8)
**Medical complaint**	ENT diseases†	9,904	4.6 (2.2-8.3)	3.8 (3.5-4.1)	4.3 (4.1-4.5)	15.8 (15.5-16.2)	15.5 (14.7-16.3)
	Pulmonary diseases†	11,628	40.6 (34.0-47.4)	31.8 (31.1-32.6)	18.4 (18-18.9)	0.1 (0.1-0.2)	0.1 (0.0-0.2)
	Cardiovascular diseases†	1,813	4.6 (2.2-8.3)	2.9 (2.7-3.2)	1.1 (1.0-1.2)	2.0 (1.8-2.1)	1.8 (1.6-2.1)
	Neurology†	2,777	23.0 (17.6-29.2)	5.4 (5.1-5.8)	3.4 (3.2-3.5)	1.7 (1.6-1.8)	0.0 (0.0-0.1)
	Digestive diseases†	17,134	2.3 (0.8-5.3)	19.9 (19.3-20.5)	24.6 (24.2-25.1)	12.8 (12.5-13.1)	1.6 (1.3-1.9)
	Urology-nephrology†	2,982	0.5 (0.0-2.5)	4.5 (4.1-4.8)	3.1 (2.9-3.3)	2.9 (2.7-3.1)	0.1 (0.0-0.2)
	Gynecology†	271	0.0 (0.0-1.7)	0.2 (0.2-0.3)	0.4 (0.4-0.5)	0.2 (0.1-0.2)	0.1 (0.0-0.2)
	Dermatology†	7,835	2.3 (0.8-5.3)	2.4 (2.2-2.7)	2.8 (2.6-3.0)	11.7 (11.4-12.0)	20.2 (19.3-21.1)
	Endocrinology-metabolism disorders†	267	0.9 (0.1-3.3)	1.1 (0.9-1.3)	0.2 (0.2-0.3)	0.0 (0.0-0.0)	0.0 (0.0-0.0)
	Infectious diseases†	11,962	5.5 (2.9-9.5)	7.2 (6.8-7.7)	13.0 (12.7-13.4)	12.2 (11.9-12.5)	15.6 (14.8-16.4) ‡
	Rheumatology-pain†	3,142	0.5 (0.0-2.5)	4.5 (4.2-4.8)	3.8 (3.6-4.0)	2.7 (2.6-2.9)	0.0 (0.0-0.0)
	Hematology†	161	0.0 (0.0-1.7)	0.9 (0.7-1.0)	0.1 (0.0-0.1)	0.0 (0.0-0.0)	0.0 (0.0-0.0)
	Poisoning†	174	0.0 (0.0-1.7)	0.4 (0.3-0.6)	0.2 (0.2-0.3)	0.0 (0.0-0.1)	0.0 (0.0-0.1)
	Others†	1,038	0.0 (0.0-1.7)	3.0 (2.8-3.3)	0.1 (0.0-0.1)	1.0 (0.9-1.1)	1.5 (1.2-1.8)
**Surgical complaint**	Head & neck trauma†	9,132	2.3 (0.8-5.3)	4.6 (4.3-5.0)	13.5 (13.2-13.9)	5.1 (4.9-5.4) ‡	19.5 (18.6-20.3)
	Upper limb trauma†	9,674	0.9 (0.1-3.3)	3.4 (3.1-3.7) ‡	6.3 (6.1-6.6)	14.4 (14-14.7)	12.3 (11.6-13.0)
	Lower limb trauma†	8,290	0.0 (0.0-1.7)	1.0 (0.9-1.2)	2.1 (1.9-2.2)	15.7 (15.4-16.1)	11.4 (10.7-12.1)
	Trunk-pelvis-urogenital trauma†	946	0.5 (0.0-2.5)	0.5 (0.4-0.7)	1.1 (1.0-1.2)	1.1 (1.0-1.3)	0.1 (0.1-0.2)
	Burns†	408	2.3 (0.8-5.3)	0.5 (0.4-0.7)	0.5 (0.4-0.6)	0.3 (0.3-0.4) ‡	0.1 (0.1-0.3)
	Others†	716	9.2 (5.7-13.9)	1.7 (1.5-1.9)	1.0 (0.9-1.1)	0.2 (0.2-0.3)	0.0 (0.0-0.0)

Values are presented as percentages with their 95% CI
†$p<0.001$ by level of triage
‡$p<0.001$ by periods: 2016-2017 versus 2018-2019

Table A3. Agreement in triage between *pediaTRI* versus PEWS in screening “high-level emergency” and “low-level emergency”

PEWS	Pediatric triage tool *pediaTRI*					
	**Stage 1 [a]**	**Stage 2 [a]**	**Stage 3 [b]**	**Stage 4 [b]**	**Stage 5 [b]**	**Total**
**≥ 4/9 [a]**	74 [c]	1,624 [c]	575 [d]	51 [d]	5 [d]	2,329
**≤ 3/9 [b]**	143 [e]	14,379 [e]	34,048 [c]	41,284 [c]	8,071 [c]	97,925
**Total**	217	16,003	34,623	41,335	8,076	100,254

[a] High-Level of emergency
[b] Low-level of emergency
[c] Agreement in triaging patients of *pediaTRI* versus PEWS according to high-level emergencies
[d] Under-triage of *pediaTRI* versus PEWS
[e] Over-triage of *pediaTRI* versus PEWS

Table A4. Sensitivity, specificity, predictive values and likelihood ratios of *pediaTRI* versus PEWS (Continued on next page)

			High-Level emergency % (CI95)*							
		**n**	***pediaTRI***	**PEWS**	***Sensitivity***	***Specificity***	***PPV***	***NPV***	***LR+***	***LR-***
**Total†**		100,254	16.2 (16.0-16.4)	2.3 (2.2-2.4)	72.9 (71.1-74.7)	85.2 (84.9-85.4)	10.5 (10.0-10.9)	99.2 (99.2-99.3)	4.9 (4.8-5.1)	0.3 (0.3-0.3)
**Sex†**	Male	54,993	17.3 (17.0-17.6)	2.4 (2.3-2.6)	73.2 (70.8-75.6)	84.1 (83.8-84.4)	10.2 (9.6-10.9)	99.2 (99.1-99.3)	4.6 (4.4-4.8)	0.3 (0.3-0.3)
	Female	45,249	14.8 (14.5-15.1)	2.2 (2.1-2.3)	72.5 (69.6-75.2)	86.5 (86.2-86.8)	10.8 (10.1-11.6)	99.3 (99.2-99.4)	5.4 (5.2-5.6)	0.3 (0.3-0.4)
**Age**	[0-28d[	1,264	94.8 (93.4-95.9) ‡	0.9 (0.4-1.6)	100.0 (71.5-100.0)	5.3 (4.1-6.7) ‡	0.9 (0.5-1.6)	100.0 (94.6-100.0)	1.1 (1.0-1.1)	NA
	[28d-3m[†	2,630	63.4 (61.5-65.2) ‡	0.9 (0.6-1.4)	95.8 (78.9-99.9)	36.9 (35.1-38.8) ‡	1.4 (0.9-2.1)	99.9 (99.4-100.0)	1.5 (1.4-1.7)	0.1 (0.0-0.1)
	[3m-1y[†	9,800	17.7 (16.9-18.4) ‡	0.5 (0.4-0.6)	83.0 (69.2-92.4)	82.6 (81.9-83.4) ‡	2.3 (1.6-3.1)	99.9 (99.8-100.0)	4.8 (4.2-5.5)	0.2 (0.1-0.4)
	[1y-3y[†	22,319	14.6 (14.1-15.1) ‡	5.8 (5.5-6.1)	70.3 (67.7-72.7)	88.9 (88.4-89.3)	28 (26.5-29.6)	98.0 (97.8-98.2)	6.3 (6.0-6.6)	0.3 (0.3-0.4)
	[3y-7y[†	26,159	12.7 (12.3-13.1)	2.3 (2.2-2.5)	76.7 (73.1-80.0)	88.8 (88.4-89.2)	14.1 (13-15.3)	99.4 (99.3-99.5)	6.9 (6.5-7.2)	0.3 (0.2-0.3)
	[7y-12y[†	21,392	11.7 (11.3-12.2)	0.6 (0.5-0.8)	74.1 (66.0-81.2)	88.7 (88.3-89.1)	4.1 (3.4-5.0)	99.8 (99.7-99.9)	6.6 (5.9-7.3)	0.3 (0.2-0.4)
	[12y-18y[†	16,690	15.2 (14.6-15.7)	1.2 (1.0-1.4)	71.1 (64.2-77.3)	85.5 (85.0-86.0)	5.5 (4.7-6.5)	99.6 (99.5-99.7)	4.9 (4.5-5.4)	0.3 (0.3-0.4)
**Type of complaint†‡**	Medical	70,909	20.1 (19.8-20.4)	3.3 (3.1-3.4)	73.1 (71.2-74.9)	81.7 (81.4-82.0)	11.9 (11.3-12.4)	98.9 (98.8-99.0)	4.0 (3.9-4.1)	0.3 (0.3-0.4)
	Surgical	29,345	6.7 (6.4-7.0)	0.0 (0.0-0.1)	46.2 (19.2-74.9)	93.3 (93.1-93.6) ‡	0.3 (0.1-0.7)	100.0 (99.9-100.0)	6.9 (3.9-12.5)	0.6 (0.3-1.0)
**Medical complaint**	ENT diseases†	9,904	6.3 (5.8-6.8)	0.2 (0.1-0.3)	43.5 (23.2-65.5)	93.8 (93.3-94.3)	1.6 (0.8-2.9)	99.9 (99.8-99.9)	7.0 (4.4-11.2)	0.6 (0.4-0.9)
	Pulmonary diseases†	11,628	44.6 (43.7-45.5)	17.2 (16.5-17.9)	75.4 (73.4-77.3)	61.8 (60.9-62.8)	29.1 (27.9-30.4)	92.4 (91.7-93.0)	2.0 (1.9-2.0)	0.4 (0.4-0.4)
	Cardiovascular diseases	1,813	26.5 (24.5-28.6) ‡	3.1 (2.4-4.1)	38.6 (26.0-52.4)	73.9 (71.8-76.0) ‡	4.6 (2.9-6.9)	97.4 (96.4-98.2)	1.5 (1.1-2.1)	0.8 (0.7-1.0)
	Neurology†	2,777	33.1 (31.4-34.9)	2.3 (1.8-3.0)	92.3 (83.0-97.5)	68.3 (66.5-70.0)	6.5 (5.0-8.3)	99.7 (99.4-99.9)	2.9 (2.7-3.2)	0.1 (0.0-0.3)
	Digestive diseases†	17,134	18.6 (18.0-19.2) ‡	0.2 (0.1-0.2)	60.7 (40.6-78.5)	81.4 (80.9-82.0) ‡	0.5 (0.3-0.9)	99.9 (99.9-100.0)	3.3 (2.4-4.4)	0.5 (0.3-0.8)
	Urology-nephrology	2,982	24.0 (22.5-25.6)	0.0 (0.0-0.1)	NA	76.0 (74.4-77.5)	0.0 (0.0-0.5)	100.0 (99.8-100.0)	NA	NA
	Gynecology	2,71	12.9 (9.2-17.5)	0.4 (0.0-2.0)	100.0 (2.5-100.0)	87.4 (82.8-91.1)	2.9 (0.1-14.9)	100.0 (98.4-100.0)	7.9 (5.8-10.9)	NA
	Dermatology	7,835	5.0 (4.5-5.5)	0.1 (0.1-0.3)	36.4 (10.9-69.2)	95.0 (94.5-95.5)	1.0 (0.3-2.6)	99.9 (99.8-100.0)	7.3 (3.3-1.6)	0.7 (0.4-1.0)
	Endocrino-metabo. Dis.	267	66.3 (60.3-71.9)	3.0 (1.3-5.8)	87.5 (47.3-99.7)	34.4 (28.6-40.5)	4.0 (1.6-8.0)	98.9 (94.0-100.0)	1.3 (1.0-1.8)	0.4 (0.1-2.3)
	Infectious diseases†	11,962	9.8 (9.3-10.3)	0.8 (0.7-1.0)	50.0 (39.8-60.2)	90.5 (90.0-91.1)	4.3 (3.2-5.6)	99.5 (99.4-99.7)	5.3 (4.3-6.5)	0.5 (0.5-0.7)
	Rheumatology-pain	3,142	22.9 (21.5-24.4)	0.2 (0.1-0.4)	83.3 (35.9-99.6)	77.2 (75.7-78.7)	0.7 (0.2-1.6)	100.0 (99.8-100.0)	3.7 (2.5-5.3)	0.3 (0.0-1.3)
	Hematology	161	85.1 (78.6-90.2)	0.6 (0.0-3.4)	100.0 (2.5-100.0)	15.0 (9.9-21.5)	0.7 (0.0-4.0)	100.0 (85.8-100.0)	1.2 (1.1-1.3)	NA
	Poisoning	174	40.8 (33.4-48.5)	1.7 (0.4-5.0)	66.7 (9.4-99.2)	59.6 (51.9-67.1)	2.8 (0.3-9.8)	99.0 (94.7-100.0)	1.7 (0.7-3.8)	0.6 (0.1-2.8)
	Others	1,038	46.5 (43.5-49.6)	0.0 (0.0-0.4)	NA	53.5 (50.4-56.5)	0.0 (0.0-0.8)	100.0 (99.3-100.0)	NA	NA
**Surgical complaint**	Head & neck trauma	9,132	8.2 (7.6-8.7) ‡	0.1 (0.0-0.2)	42.9 (9.9-81.6)	91.9 (91.3-92.4)	0.4 (0.1-1.2)	100.0 (99.9-100.0)	5.3 (2.2-12.4)	0.6 (0.3-1.2)
	Upper limb trauma	9,674	5.7 (5.2-6.2) ‡	0.1 (0.0-0.1)	40.0 (5.3-85.3)	94.3 (93.9-94.8) ‡	0.4 (0.0-1.3)	100.0 (99.9-100.0)	7.1 (0.2-20.8)	0.6 (0.3-1.3)
	Lower limb trauma	8,290	2.0 (1.7-2.3)	0.0 (0.0-0.0)	NA	98.0 (97.7-98.3)	0.0 (0.0-2.3)	100.0 (100.0-100.0)	NA	NA
	Trunk-pelv-urogen. trauma	946	9.3 (7.5-11.3)	0.0 (0.0-0.4)	NA	90.7 (88.7-92.5)	0.0 (0.0-4.1)	100.0 (99.6-100.0)	NA	NA
	Burns	408	22.1 (18.1-26.4)	0.0 (0.0-0.9)	NA	77.9 (73.6-81.9)	0.0 (0.0-4.0)	100.0 (98.8-100.0)	NA	NA
	Others	716	39.9 (36.3-43.6)	1.5 (0.8-2.7)	36.4 (10.9-69.2)	60.0 (56.3-63.6)	1.4 (0.4-3.5)	98.4 (96.7-99.3)	0.9 (0.4-2.0)	1.1 (0.7-1.7)

*Immediate and very urgent category; LR+=likelihood ratio for high-level emergency triage test result; LR−=likelihood ratio for low-level emergency triage test result.
Sensitivity % (CI95)= high-level emergency; Specificity % (CI95)= low-level emergency
†p<0.001 per type of level of emergency assigned by pediaTRI versus PEWS; ‡p<0.001 by periods: 2016-2017 versus 2018-2019; NA Not applicable

Table A4. Sensitivity, specificity, predictive values and likelihood ratios of *pediaTRI* versus PEWS (continued from previous page)

			High-Level emergency % (CI95)*							
		**n**	***pediaTRI***	**PEWS**	**Sensitivity**	**Specificity**	**PPV**	**NPV**	**LR+**	**LR-**
**Diagnosis**	ENT infect. Dis.†	15,108	6.6 (6.2-7.0)	1.5 (1.4-1.8)	27.5 (21.8-33.7)	93.8 (93.4-94.2)	6.5 (5.0-8.2)	98.8 (98.6-99.0)	4.4 (3.5-5.5)	0.8 (0.7-0.8)
	Acute gastroenteritis†	8,502	19.5 (18.6-20.3) ‡	0.4 (0.3-0.5)	57.6 (39.2-74.5)	80.7 (79.8-81.5) ‡	1.1 (0.7-1.8)	99.8 (99.7-99.9)	3.0 (2.2-4.0)	0.5 (0.4-0.8)
	Asthma†	3,073	55.8 (54.0-57.5)	37.1 (35.4-38.9)	86.3 (84.2-88.3)	62.3 (60.1-64.4) ‡	57.5 (55.1-59.8)	88.5 (86.7-90.2)	2.3 (2.2-2.4)	0.2 (0.2-0.3)
	Bronchiolitis†	1,682	60.8 (58.4-63.1) ‡	10.1 (8.7-11.6)	77.1 (70.0-83.1)	41.1 (38.6-43.6)	12.8 (10.8-15.0)	94.1 (92.0-95.8)	1.3 (1.2-1.4)	0.6 (0.4-0.7)
	Flu†	1,080	15.5 (13.4-17.8)	2.7 (1.8-3.8)	48.3 (29.4-67.5)	85.4 (83.2-87.5)	8.4 (4.7-13.7)	98.4 (97.3-99.1)	3.3 (2.2-5.0)	0.6 (0.4-0.9)
	Fever†	5,044	14.4 (13.5-15.4)	2.0 (1.6-2.4)	51.5 (41.3-61.6)	86.3 (85.4-87.3)	7.2 (5.4-9.3)	98.9 (98.5-99.2)	3.8 (3.1-4.6)	0.6 (0.5-0.7)
	Mild trauma brain injury	5,238	10.3 (9.4-11.1)	0.1 (0.0-0.2)	50.0 (6.8-93.2)	89.8 (88.9-90.6)	0.4 (0.0-1.3)	100.0 (99.8-100.0)	4.9 (1.8-13.1)	0.6 (0.2-1.5)
	Upper limb trauma	9,702	7.1 (6.6-7.7)	0.1 (0.0-0.1)	50.0 (11.8-88.2)	92.9 (92.4-93.4)	0.4 (0.1-1.3)	100.0 (99.9-100.0)	7.0 (3.2-15.7)	0.5 (0.2-1.2)
	Lower limb trauma	8,171	2.8 (2.5-3.2)	0.0 (0.0-0.1)	100.0 (2.5-100.0)	97.2 (96.8-97.5)	0.4 (0.0-2.4)	100.0 (100.0-100.0)	3.6 (3.1-4.0)	NA
	Burn	453	21.9 (18.1-25.9)	0.0 (0.0-0.8)	NA	78.1 (74.1-81.9)	0.0 (0.0-3.7)	100.0 (99.0-100.0)	NA	NA
	Visceral pathologies	1,297	42.3 (39.5-45.0)	0.2 (0.0-0.6)	50.0 (1.3-98.7)	57.8 (55.0-60.5)	0.2 (0.0-1.0)	99.9 (99.3-100.0)	1.2 (0.3-4.7)	0.9 (0.2-3.5)

*Immediate and very urgent category; LR+=likelihood ratio for high-level emergency triage test result; LR−=likelihood ratio for low-level emergency triage test result.
Sensitivity % (CI95)= high-level emergency; Specificity % (CI95)= low-level emergency
†p<0.001 per type of level of emergency assigned by pediaTRI versus PEWS; ‡p<0.001 by periods: 2016-2017 versus 2018-2019; NA Not applicable
