# Supplementary material for: Validation of the Computerized Pediatric Triage Tool, pediaTRI, in the Pediatric Emergency Department of Lenval Children's Hospital in Nice: A Cross-Sectional Observational Study
Source: Front Pediatr. 2022 Apr 26;10:840181. doi: 10.3389/fped.2022.840181 (PMC9113392; doi:10.3389/fped.2022.840181)
Supplement: Supplementary file 3 [file Data_Sheet_3.pdf]

Appendix 3. Percentages of patients by level of triage in the literature

Etude	Sample size	Triage tool and objectives	Design	Level 1	Level 2	Level 3	Level 4	Level 5
**Our study** 2017, *France*	100,506	**pediaTRI** versus PEWS ≥ 4	Cross-sectional	0.2 (0.2_0.2)	16.5 [16.3-16.7]	34.7 {34.4-35.0)	41.1 [40.8-41.4)	7.5 [7.4-7.7]
**Roukema** 2006, *Netherlands*	1,065	**MTS** Versus Gold standard (based on the use of resources and expert committee)	Retrospective	11.9 (10.0-14.0)	25.9 (23.3-28.7)	25.5 (22.9-28.2)	26.7 (24.0-29.4)	10.1 (8.3-12.0)
**Van Veen** 2008, *Netherlands*	13,554	**MTS** Versus Gold standard (based on the use of resources and expert committee)	Cross-sectional	1.5 (1.3-1.7)	21.4 (20.7)	36.2 (35.3-37.0)	40.1 (39.3-41.0)	0.8 (0.7-1.0)
**Van Veen** 2012, *Netherlands*	11,260	**MTS** Versus Gold standard (based on the use of resources and expert committee)	Cross-sectional	2.1 (1.8-2.4)	14.1 (13.4-14.7)	36.2 (35.3-37.1)	44.2 (43.3-45.1)	3.5 (3.2-3.8)
**Seiger** 2013, *Netherlands*	n1=2,960 (with chronic disease) n2=5,632 (without chronic disease)	**MTS** Versus Gold standard (based on the use of resources and expert committee)	Cross-sectional	(1) 3.6 (2.9-4.3) (2) 3.0 (2.6-3.5)	(1) 24.1 (22.6—25.7) (2) 26.4 (25.2-27.5)	(1) 55.0 (53.2-56.8) (2) 45.5 (44.2-46.8)	(1) 17.2 (15.8-18.6) (2) 24.6 (23.5-25.8)	(1) 0.1 (0.0-0.3) (2) 0.5 (0.4-0.8)
**Seiger** 2014, *Netherlands, Portugal, United Kingdom*	60,735	**(1) MTS original** **(2) MTS version 1** versus hospitalization	Cross-sectional	1.3 (1.2-1.4)	15.84 (15.6-16.1)	23.3 (23.0-23.6)	59.6 (59.2-60.0)	
**Travers** 2009, *USA*	1,173	**ESI** versus resource utilization	Cross-sectional	16.5 (14.5-18.8)	21.3 (19.0-23.8)	20.0 (17.8-22.4)	21.8 (19.5-24.3)	20.3 (18.0-22.7)
**Green** 2012, USA	780	**ESI** versus resource utilization	Retrospective	0.3 (0.0-0.9)	9.4 (7.4-11.6)	37.1 (33.7-40.6)	32.2 (28.9-35.6)	21.2 (18.3-24.2)
**Baumann** 2005, *USA*	510	**ESI** versus resource utilization	Cross-sectional	2.9 (1.7-4.8)	18.6 (15.3-22.3)	34.12 (30.0-38.4)	36.7 (32.5-41.0)	7.7 (5.5-10.3)
**Gouin** 2005, *Canada*	537	**PedCTAS** versus resource utilization /PRISA score	Cross-sectional	0.4 (0.1-1.3)	4.7 (3.0-6.8)	48.4 (44.1-52.7)	37.1 (33.0-41.3)	9.5 (7.2-12.3)
**Warren** 2008, *Canada*	1,618	**PedCTAS** versus resource utilization	Retrospective	0.4 (0.1-0.8)	9.6 (8.2-11.1)	48.0 (45.5-50.4)	38.8 (36.4-41.2)	3.3 (2.5-4.3)
**Gravel** 2008, *Canada*	19,265	**PedCTAS** and reliability study	Cross-sectional	1.5 (1.4-1.7)	5.2 (4.8-5.5)	28.5 (27.9-29.1)	49.5 (48.8-50.2)	15.4 (14.9-15.9)
**Gravel** 2013, *Canada*	550,940	**PedCTAS** versus resource utilization	Retrospective	0.6 (0.6-0.7)	11.3 (11.3-11.4)	37.5 (37.4-37.6)	43.6 (43.5-43.8)	6.6 (6.6-6.7)
**Gravel** 2012, *Canada*	395,661	**PedCTAS** versus resource utilization, reliability study	Cross-sectional	0.7 (0.7-0.7)	12.5 (12.4-12.6)	41.2 (41.0-41.3)	41.8 (41.7-42.0)	0.4 (0.4-0.4)
**Gravel** 2009, *Canada*	58,529	**PedCTAS** versus resource utilization	Retrospective	1.2 (1.2-1.3)	6.8 (6.6-7.0)	31.5 (31.2-31.9)	46.9 (46.5-47.3)	13.5 (13.3-13.8)
**Gaucher** (1)2010, *Canada*	60,525	**PedCTAS,** to assess the characteristics of patients who left a pediatric ED without being seen by a physician	Retrospective	0.9 (0.8-1.0)	7.7 (7.4-7.9)	31.0 (30.7-31.4)	50.1 (49.7-50.5)	10.6 (10.4-10.9)
**Acworth** 2009, *Australia – New-Zealand*	350,345	**ATS,** to describe epidemiological data concerning paediatric ED visits to an Australian and New Zealand research network	Cross-sectional	0.6 (0.6-0.6)	4.5 (4.4-4.5)	27.4 (27.3-27.6)	52.1 (51.9-52.3)	15.4 (15.3-15.6)

Roukema J. Steyerberg EW. van Meurs A. Ruige M. van der Lei J. Moll HA. Validity of the Manchester Triage System in paediatric emergency care. Emerg Med J. 2006;23(12):906–10.

van Veen M. Steyerberg EW. Ruige M. van Meurs AH. Roukema J. van der Lei J. et al. Manchester triage system in paediatric emergency care: prospective observational study. BMJ. 2008;337:a1501

van Veen M. Steyerberg EW. Van't Klooster M. Ruige M. van Meurs AH. van der Lei J. et al. The Manchester triage system: improvements for paediatric emergency care. Emerg Med J. 2012;29(8):654–9.

Seiger N. van Veen M. Steyerberg EW. et al. Accuracy of triage for children with chronic illness and infectious symptoms. Pediatrics 2013;132:e1602–e1608.

Seiger N. van Veen M. Almeida H. et al. Improving the Manchester triage system for pediatric emergency care: an international multicenter study. PLoS One 2014;9:e83267.

Travers DA, Waller AE, Katznelson J, Agans R. Reliability and validity of the emergency severity index for pediatric triage. Acad Emerg Med Off J Soc Acad Emerg Med. 2009 Sep;16(9):843–9.

Green NA, Durani Y, Brecher D, DePiero A, Loiselle J, Attia M. Emergency Severity Index version 4: a valid and reliable tool in pediatric emergency department triage. Pediatr Emerg Care. 2012 Aug;28(8):753–7.

Baumann MR, Strout TD. Evaluation of the Emergency Severity Index (version 3) triage algorithm in pediatric patients. Acad Emerg Med Off J Soc Acad Emerg Med. 2005 Mar;12(3):219–24.

Gouin S, Gravel J, Amre DK, Bergeron S. Evaluation of the Paediatric Canadian Triage and Acuity Scale in a pediatric ED. Am J Emerg Med. 2005 May;23(3):243–7.

Warren DW, Jarvis A, LeBlanc L, Gravel J, CTAS National Working Group, Canadian Association of Emergency Physicians, et al. Revisions to the Canadian Triage and Acuity Scale paediatric guidelines (PaedCTAS). CJEM. 2008 May;10(3):224–43.

Gravel J, Gouin S, Manzano S, Arsenault M, Amre D. Interrater agreement between nurses for the Pediatric Canadian Triage and Acuity Scale in a tertiary care center. Acad Emerg Med Off J Soc Acad Emerg Med. 2008 Dec;15(12):1262–7.

Gravel J, Fitzpatrick E, Gouin S, Millar K, Curtis S, Joubert G, et al. Performance of the Canadian Triage and Acuity Scale for children: a multicenter database study. Ann Emerg Med. 2013 Jan;61(1):27-32.e3.

Gravel J, Gouin S, Goldman RD, Osmond MH, Fitzpatrick E, Boutis K, et al. The Canadian Triage and Acuity Scale for children: a prospective multicenter evaluation. Ann Emerg Med. 2012 Jul;60(1):71-77.e3.

Gravel J, Manzano S, Arsenault M. Validity of the Canadian Paediatric Triage and Acuity Scale in a tertiary care hospital. CJEM. 2009 Jan;11(1):23–8.

Gaucher N, Bailey B, Gravel J. Who are the children leaving the emergency department without being seen by a physician? Acad Emerg Med Off J Soc Acad Emerg Med. 2011 Feb;18(2):152–7.

Acworth J, Babl F, Borland M, Ngo P, Krieser D, Schutz J, et al. Patterns of presentation to the Australian and New Zealand Paediatric Emergency Research Network. Emerg Med Australas EMA. 2009 Feb;21(1):59–66.
