# Supplementary material for: Validation of the Computerized Pediatric Triage Tool, pediaTRI, in the Pediatric Emergency Department of Lenval Children's Hospital in Nice: A Cross-Sectional Observational Study
Source: Front Pediatr. 2022 Apr 26;10:840181. doi: 10.3389/fped.2022.840181 (PMC9113392; doi:10.3389/fped.2022.840181)
Supplement: Supplementary file 4 [file Data_Sheet_4.pdf]

Appendix 4. Performance of pediatric triage systems in emergency care

Study	Sample size	Triage system	Design	Outcome	Performance	Conclusion
**Roukema 2006** *Netherlands*	n = 1,065	**MTS** Versus Gold standard (based on resource utilization and expert committee)	Retrospective	Screening high-level emergency (level 1 and 2)	Se 63%, Sp 78%<br>under-triage 15%, over-triage 40%	Moderate sensitivity and specificity
**Van Veen 2008** *Netherlands*	n = 13,554	**MTS** Versus Gold standard (based on resource utilization and expert committee)	Cross-sectional	Screening high-level emergency (level 1 and 2)	Se 63% (59-66), Sp 79% (79-80)<br>under-triage 12%, over-triage 54%<br>LR+ 3 (2.8-3.2), LR- 0.5 (0.4-0.5)	Moderate sensitivity and specificity
**Van der Wulp 2008** *Netherlands*	11 scenarios	**MTS** Versus Gold standard (based on resource utilization and expert committee)	Cross-sectional	Screening high-level emergency (level 1 and 2)	Se 83.3%, Sp 93.7%<br>under-triage 24.7%, over-triage 8.2%	Good sensitivity and specificity
**Van Veen 2012** *Netherlands*	n = 11,260	**MTS** Versus Gold standard (based on resource utilization and expert committee)	Cross-sectional	Screening high-level emergency (level 1 and 2)	Se 64% (60-68), Sp 87% (86-87)<br>under-triage 15%, over-triage 47.7%	Moderate sensitivity and good specificity
**Aiemchanbanjong 2017** *Thailand*	n = 1,041	**ATS, PedCTAS MTS, ESI** Versus Gold standard (based on resource utilization and expert committee)	Cross-sectional	Screening high-level emergency (level 1 and 2)	**ATS** : Se 13%, Sp 94%, AUC 0.7 (0.7-0.8)<br>**CTAS** : Se 50%, Sp 74%, AUC 0.6 (0.6-0.7)<br>**MTS** : Se 57%, Sp 69%, AUC 0.70 (0.7-0.7)<br>**ESI** : Se 52%, Sp 81%, AUC 0.78 (0.7-0.8)	ESI more performant than other triage systems
**Seiger 2013** *Netherlands*	n1 = 2,960 with chronic diseases<br>n2 = 5,632 without chronic diseases	**MTS** Versus Gold standard (based on resource utilization and expert committee)	Cross-sectional	Screening high-level emergency (level 1 and 2)	Se1 58% (53-62), Sp1 78% (76-79)<br>LR+1 2.6 (2.4-2.9), LR-1 0.7 (0.7-0.7)<br>under-triage1 17% over-triage1 48%<br><br>Se2 74% (70-78), Sp2 75% (74-77)<br>LR+2 1.1 (1.1-1.2), LR-2 0.8 (0.7-0.9)<br>under-triage2 11%, over-triage2 59%	Moderate performance, Lower performance with patients affected by chronic diseases
**Seiger 2014** *Netherlands Portugal United Kingdom*	n = 60,375	**(1) MTS original**<br>**(2) MTS version 1** Versus hospitalization	Cross-sectional	Hospitalization rate	LR+1 3.2 (3.0-3.3), LR-1 0.7 (0.6-0.7)<br>LR+2 4.3 (4.1-4.4), LR-2: 0.7 (0.7-0.70)	Performance of MTSv1 better than original MTS
**Nijman 2011** *Netherlands*	n = 1,911	**MTS** Versus severe infectious diseases	Cross-sectional	Screening severe infectious diseases	Se 42% (35-41), Sp 69 (66-72)<br>LR+1.4(1.1-1.7), LR-0.8 (0.7-1.0)	Lower performance with patients affected by severe infectious diseases
**Our study** *France*	n = 100,506	***pediaTRI*** Versus PEWS ≥ 4	Cross-sectional	Screening high-level emergency (level 1 and 2)	Se 76.4% [74.6-78.2], Sp 84.7% [84.4-84.9]<br>under-triage 0.5% (0.5-0.6),<br>over-triage 15.0% (14.8-15.2)<br>LR+ 5.0 (4.8-5.1), LR- 0.3 (0.3-0.3)	Moderate sensitivity and good specificity

Roukema J, Steyerberg EW, van Meurs A, Ruige M, van der Lei J, Moll HA. Validity of the Manchester Triage System in paediatric emergency care. Emerg Med J. 2006;23(12):906–10.

van Veen M, Steyerberg EW, Ruige M, van Meurs AH, Roukema J, van der Lei J, et al. Manchester triage system in paediatric emergency care: prospective observational study. BMJ. 2008;337:a1501

van der WI, van Baar ME, Schrijvers AJ. Reliability and validity of the Manchester Triage System in a general emergency department patient population in the Netherlands: results of a simulation study. Emerg Med J. 2008;25(7):431–4.

van Veen M, Steyerberg EW, Van't Klooster M, Ruige M, van Meurs AH, van der Lei J, et al. The Manchester triage system: improvements for paediatric emergency care. Emerg Med J. 2012;29(8):654–9.

Aeimchanbanjong K, Pandee U. Validation of different pediatric triage systems in the emergency department. World J Emerg Med. 2017;8(3):223-227.

Seiger N, van Veen M, Steyerberg EW, et al. Accuracy of triage for children with chronic illness and infectious symptoms. Pediatrics 2013;132:e1602–e1608.

Seiger N, van Veen M, Almeida H, et al. Improving the Manchester triage system for pediatric emergency care: an international multicenter study. PLoS One 2014;9:e83267.

Nijman RG, Zwinkels RL, van Veen M, et al. Can urgency classi cation of the Manchester triage system predict serious bacterial infections in febrile children? Arch Dis Child 2011;96:715–22.
