# Supplementary material for: Validation of the Computerized Pediatric Triage Tool, pediaTRI, in the Pediatric Emergency Department of Lenval Children's Hospital in Nice: A Cross-Sectional Observational Study
Source: Front Pediatr. 2022 Apr 26;10:840181. doi: 10.3389/fped.2022.840181 (PMC9113392; doi:10.3389/fped.2022.840181)
Supplement: Supplementary file 5 [file Data_Sheet_5.pdf]

Appendix 5. Prevalence of main diagnosis encountered at the PED

<table>
<tr><th>Study</th><th>n patients</th><th>Bronchiolitis</th><th>Asthma</th><th>Acute gastroenteritis</th><th>ENT infectious diseases</th><th>Influenza</th><th>Limb fracture/Trauma</th><th>Brain injury</th></tr>
<tr><td>Our study</td><td>100,506</td><td>n=1,654
1.6 (1.6-1.7)</td><td>n=2,856
2.8 (2.7-2.9)</td><td>n=9,002
9.0 (8.8-9.1)</td><td>n=15,978
15.9 (15.7-16.1)</td><td>n=1,669
1.7 (1.5-1.7)</td><td>n=18,464
18.4 (18.1-18.6)</td><td>n=5,400
5.4 (5.2-5.5)</td></tr>
<tr><td>Gravel 2012
Canada</td><td>1,464</td><td></td><td>n=37
2.5 (1.8-3.5)</td><td colspan="3">n=142 (bact) + 314(viral)
31.2 (28.8-33.6)</td><td>n=85
5.8 (4.7-7.1)</td><td></td></tr>
<tr><td>Acworth 2009
Australie – Nouvelle Zélande</td><td>284,327</td><td>n=6,214
2.2 (2.1-2.2)</td><td>n=9,598
3.4 (3.3-3.4)</td><td>n=18,094
6.4 (6.3-6.5)</td><td colspan="2">n=17422
6.1 (6.0-6.2)</td><td>n=4,015
1.4 (1.4-1.5)</td><td></td></tr>
<tr><td>Thakker 1994
Royaume uni</td><td>894</td><td>n=48
5.4 (4.0-7.1)</td><td>n=144
16.1 (13.8-18.7)</td><td>n=44
4.9 (3.6-6.6)</td><td></td><td></td><td></td><td></td></tr>
<tr><td>Christoffel 1985
Etats-Unis</td><td>7,844</td><td>n=86
1.1 (0.9-1.4)</td><td>n=455
5.8 (5.3-6.3)</td><td>n=416
5.3 (4.8-5.8)</td><td></td><td></td><td></td><td>n=180
2.3 (2.0-2.7)</td></tr>
</table>

Gravel J, Gouin S, Goldman RD, Osmond MH, Fitzpatrick E, Boutis K, et al. The Canadian Triage and Acuity Scale for children: a prospective multicenter evaluation. Ann Emerg Med. 2012 Jul;60(1):71-77.e3.

Acworth J, Babl F, Borland M, Ngo P, Krieser D, Schutz J, et al. Patterns of presentation to the Australian and New Zealand Paediatric Emergency Research Network. Emerg Med Australas EMA. 2009 Feb;21(1):59–66.

Thakker Y, Sheldon TA, Long R, MacFaul R. Paediatric inpatient utilisation in a district general hospital. Arch Dis Child. 1994 Jun;70(6):488–92.

Christoffel KK, Garside D, Tokich T. Pediatric emergency department utilization in the 1970s. Am J Emerg Med. 1985 May;3(3):177–81.
